# Hexaarylbenzene based high-performance p-channel molecules for electronic applications

**DOI:** 10.1039/d1ra00217a

**Published:** 2021-03-22

**Authors:** Panneerselvam Devibala, Balu Balambiga, Shana Noureen, Samuthira Nagarajan

**Affiliations:** Organic Electronics Division, Department of Chemistry, Central University of Tamil Nadu Thiruvarur 610 005 India snagarajan@cutn.ac.in

## Abstract

Hexaarylbenzene-based molecules find potential applications in organic electronics due to wider energy gap, high HOMO level, higher photoconductivity, electron-rich nature, and high hole-transporting property. Due to the unique propeller structure, these molecules show low susceptibility towards self-aggregation. This property can be tailored by proper molecular engineering by the incorporation of appropriate groups. Therefore, hexaarylbenzene chromophores are widely used as the materials for high-efficiency light-emitting materials, charge transport materials, host materials, redox materials, photochemical switches, and molecular receptors. This review highlights the diverse structural modification techniques used for the synthesis of symmetrical and unsymmetrical structures. Also, the potential applications of these molecules in organic light-emitting diodes, organic field-effect transistors, organic photovoltaics, organic memory devices, and logic circuits are discussed.

## Abstract 2

The latest progress on semiconducting applications of hexaarylbenzene is reviewed, including a fundamental overview of geometry, synthetic methods, structure-property relationship, design strategies and electronic applications in OFET, OLED and OPV.

## Graphical abstract

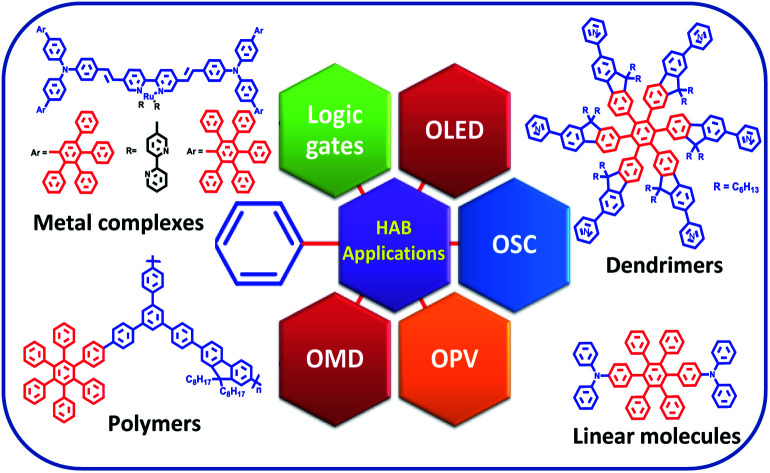

## 1. Introduction

Hexaarylbenzenes (HAB) have a characteristic propeller structure due to the steric interaction between the easily rotatable peripheral aromatic units.^1,2^ This non-centrosymmetric molecule adopts a nearly six-fold symmetry with the peripheral rings twisted to the central benzene by 25°.^3^ The resultant non-planar structure leads to weak intermolecular C–H⋯π and π–π interactions and limited intramolecular conjugation.^4^ These interactions result in a low degree of self-aggregation, wider highest occupied molecular orbital (HOMO)-lowest unoccupied molecular orbital (LUMO) energy gap, less efficient crystal packing, and higher solubility of HAB derivatives compared to planar molecules. Thus, HAB derivatives are excellent candidates for applications that require less efficient packing and low molecular cohesion.^5,6^ These interactions can be altered by introducing appropriate substitutions in the peripheral rings, which results in systematic changes in the solubility, thermal behavior, molecular cohesion, and crystal packing interactions.^4^ For instance, the substitution of flexible chains on the HAB core generates an ordered columnar mesomorphic phase which results in liquid crystalline materials.^7,8^ On the other hand, nano-aggregates were generated by the introduction of amphiphilic character to the HAB core.^9,10^ Thus, the π-extended, propeller-shaped newly functionalized HAB derivatives have exclusive applications in microporous organic solids, liquid crystals, molecular rotors, molecular capsules, redox materials, linear optics, synthetic graphene, and chemo-sensors.^11–14^ Besides, HAB derivatives are utilized as important intermediates for creating graphene architecture and hexabenzocoronene (HBC) derivatives, which are strong candidates for organic electronic applications.^15^

Unique properties of HAB have stimulated researchers from many areas such as supramolecular chemistry, bio-medical, environmental sensing, material chemistry, synthetic and organometallic chemistry.^8,16–21^ Since the pioneering work of Dilthey in 1933 on the synthesis of hexaphenylbenzene (HPB) through Diels–Alder cycloaddition reaction, polyaromatic π-systems have been the subject of tremendous exploration.^22^ Among the reported multiply substituted aromatic compounds, HPBs are highly significant due to their structural diversity and optical properties.^23,24^ Studies on the non-planar configuration, crystallographic studies, and synthetic optimization of HPBs have attracted chemists since the last century.^2,3,25^ Over the past three decades, many newly functionalized HPB molecules have been synthesized and utilized in various fields.^26^

Recently, the radially substituted HAB derivatives have been utilized as organic semiconductors for applications in organic electronics and material science. The extended conjugation of HAB derivatives raises the HOMO level, which effectively increases the electron-donating ability and hole injection/transport. It also contributes to the construction of a 3D network by confining intermolecular aggregation and creating uniform films.^27,28^ HABs with suitable electron-withdrawing and/or donating groups alter the HOMO energy level, resulting in the enhancement of electron/hole transport and emission behavior, and thus could be potentially used to establish new types of electronic devices.^29,30^ Over the past two decades, there have been immense developments to explore new types of organic semiconductors with favorable photophysical and electronic properties for optoelectronic and electronic devices.^31–33^ These organic materials replaced the inorganic counterparts due to their high flexibility, lightweight, low fabrication cost, and efficient fabrication over large areas.^34–36^ The development of organic semiconductors has remarkably improved the development of electronic devices such as organic light-emitting diodes (OLED), organic field-effect transistors (OFET), organic photovoltaics (OPV), memory devices, sequential logic circuits, radio-frequency identification (RFID) tags, sensors, organic lasers, and wearable electronics.^37–44^ Besides, new conceptual designs incorporated with higher flexibility and artificial intelligence are being introduced in the field of consumer electronics.

The performance of highly efficient organic electronic devices mainly relies on the device architecture as well as the “structure–property relationship”. This can be helpful to find an ideal material with favorable electronic energy levels and molecular interactions for application in organic electronic devices.^45^ The bulk properties such as energy gap, electron affinity, solubility as well as stability in ambient condition can be altered by modifying the structure of the organic material.^26^ The improvement of molecular architecture involves the optimization of individual molecules and control of film morphology. The desirable intramolecular and intermolecular interactions are achieved by altering the molecular system, resulting in the formation of various architectures ranging from small linear structures to dendritic or polymeric chains.^26,46^ For instance, the molecular packing and connectivity of HPB derivatives depend on the position of substitution and symmetry of the molecule.^47^ Furthermore, the introduction of electron donors and/or acceptors influences the photophysical properties, along with molecular packing and charge carrier properties. The π-extended organic small molecules and polymers are important classes of materials in modern technology due to their cost-effectiveness and convenient chemical modifications.^48–50^ Also, the monodispersity and high purity of small molecules promote the generation of uniform ordered films. The HAB-based small molecules, polymers, and dendrimers have been utilized as active materials in organic electronic devices.^26,51–53^

In this review, the applications of HAB-based molecules as semiconductors are presented to elucidate their importance in the fabrication of organic electronic devices. The synthetic challenges in the development of structural diversity within the HAB molecules were also discussed. Besides, the effect of different substituents on altering the intermolecular interactions in the solid as well as solution states upon the manipulation of molecular architecture and connectivity has been presented. The construction of various HAB-based structures is discussed along with charge transfer processes and the optical and electronic properties for the development of organic semiconductors. Besides, the performance of these molecular segments in organic electronic devices is presented.

## 2. Functionalization of hexaarylbenzene

The photophysical and electronic properties of HAB and HPBs are important for the design of organic photovoltaics, organic light-emitting diodes, and other electronic applications. Much effort is being devoted to the progress of novel synthetic strategies for the preparation of HPB derivatives as they are versatile building blocks in organic synthesis with a broad spectrum of applications.^26,47,54^ Hexaphenylbenzene was traditionally synthesized *via* three methods (i) transition metal-catalyzed cyclotrimerization reaction, (ii) Diels–Alder reaction, and (iii) C–C coupling reactions.^55^

### 2.1. Transition metal-catalyzed cyclotrimerization reaction

**Fig. 1:**
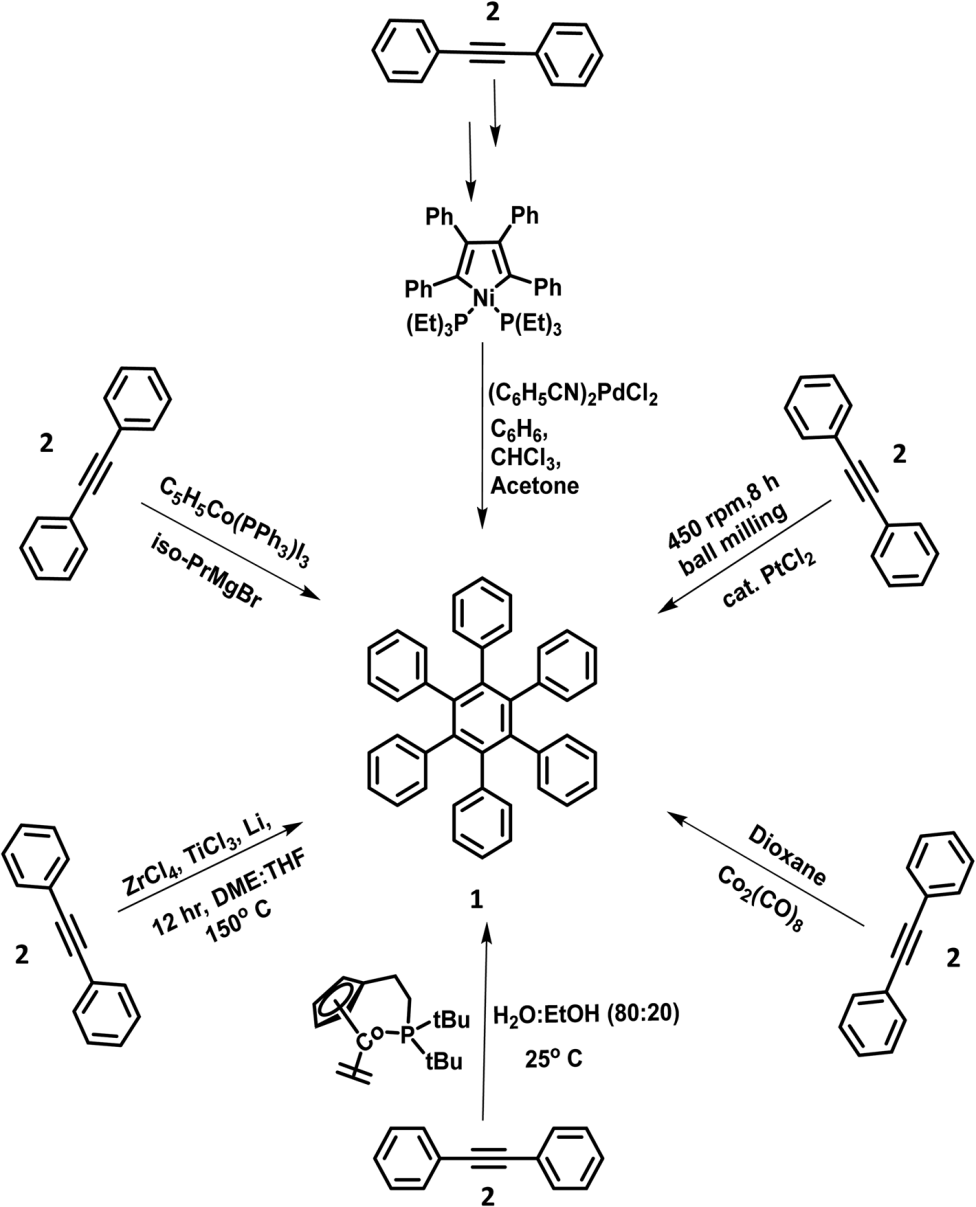
Scheme of cyclotrimerization reactions.

The metal-catalyzed [2 + 2 + 2] cyclotrimerization reactions of suitably substituted diaryl acetylenes were often performed for the synthesis of highly symmetric star-shaped (*C*^6^ and *C*^3^-symmetry) HABs.^56–58^ This synthetic strategy is advantageous due to its high yields and fewer synthetic steps involved in the synthesis. The synthesis of HPB molecules was attempted in the mid-twentieth century by adding catalytic amounts of triphenylphosphinecyclopentadienylcobaltdiiodide(ii) and isopropylmagnesium bromide to diphenylacetylene (2).^59^ However, the poor yield and higher number of synthetic steps involved in the synthesis of the desired HPB derivatives indicate the inefficiency of the Co and Mg catalyzed synthetic routes. Another possible trimerization reaction to produce HPB with good yield involves the addition of catalytic amounts of bis-(benzonitri1e)-palladium chloride(ii) to compound 2.^60^ Towards the end of the century, Ni, activated Zr–Ti, and other transition metal-promoted trimerization reactions for the synthesis of HPB were reported.^61–64^ In the 21st century, Butenschön and team^65^ reported the first Co-catalyzed cyclotrimerization of alkynes in an aqueous medium (Fig. 1). The reaction proceeded in the aqueous medium composed of water and ethanol in the ratio of 80 : 20, which resulted in the formation of 1 in higher yields. Also, the water/ethanol solvent system showed the advantages of easy waste disposal and safety compared to organic solvents.

**Fig. 2:**
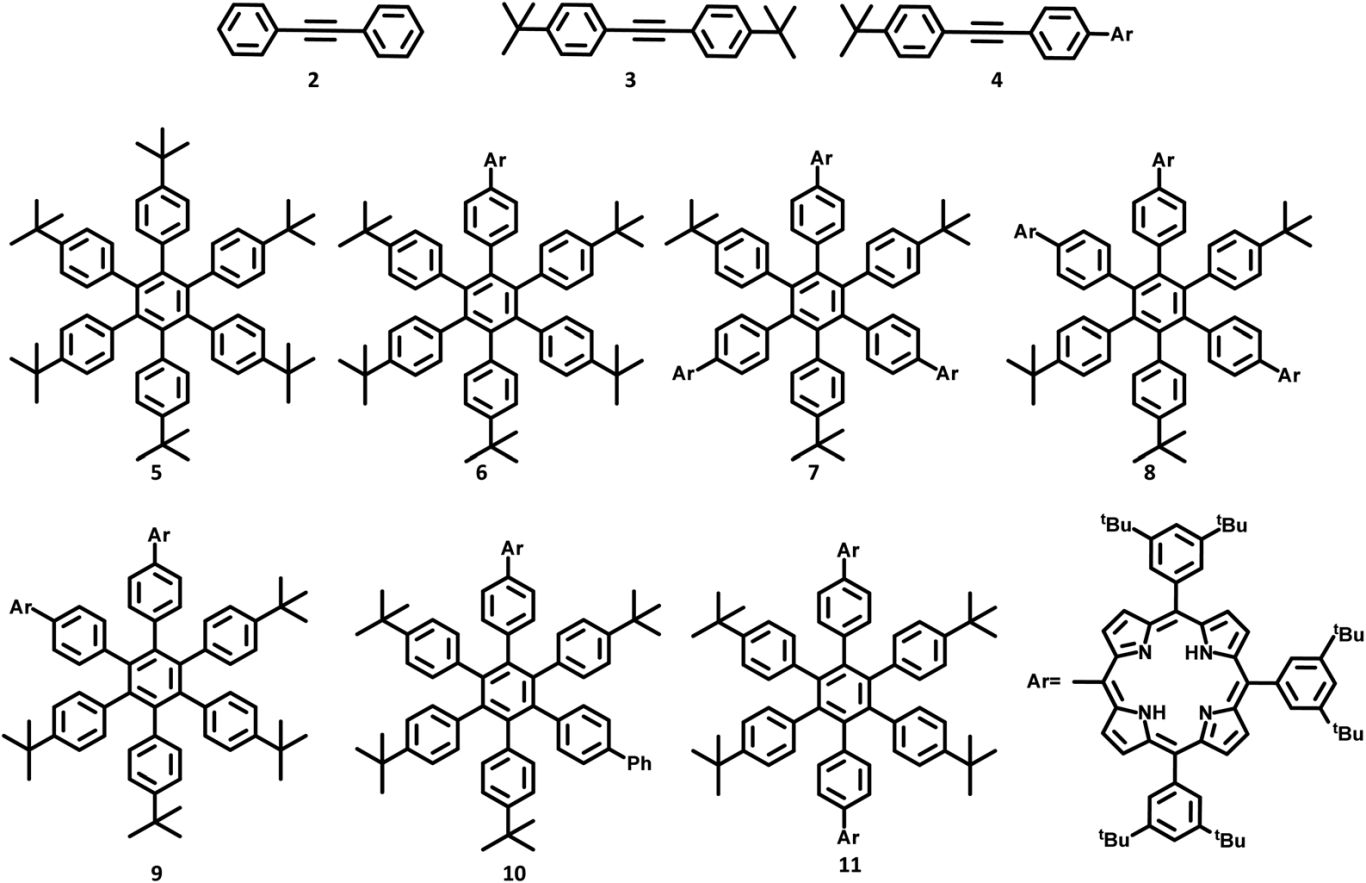
HAB derivatives 5–11 and acetylenes 2–4.

The lower-symmetric HPB derivatives can be synthesized *via* mixed cyclotrimerization of less symmetric arylacetylenes, using dicobaltoctacarbonyl as the catalyst. Recently, meso connected porphyrins containing HPB scaffolds 5–11 were reported by Martin and co-workers^66^ on the mixed cyclotrimerization reaction of 3 and 4 (Fig. 2). They have analyzed the trimerization reaction of two different arylacetylenes, among which one is symmetric while the other is asymmetric. The reaction yielded six different porphyrins appended hexasubstituted HPB derivatives (5–11) as the byproduct.

### 2.2. Diels–Alder cycloaddition reaction

**Fig. 3:**
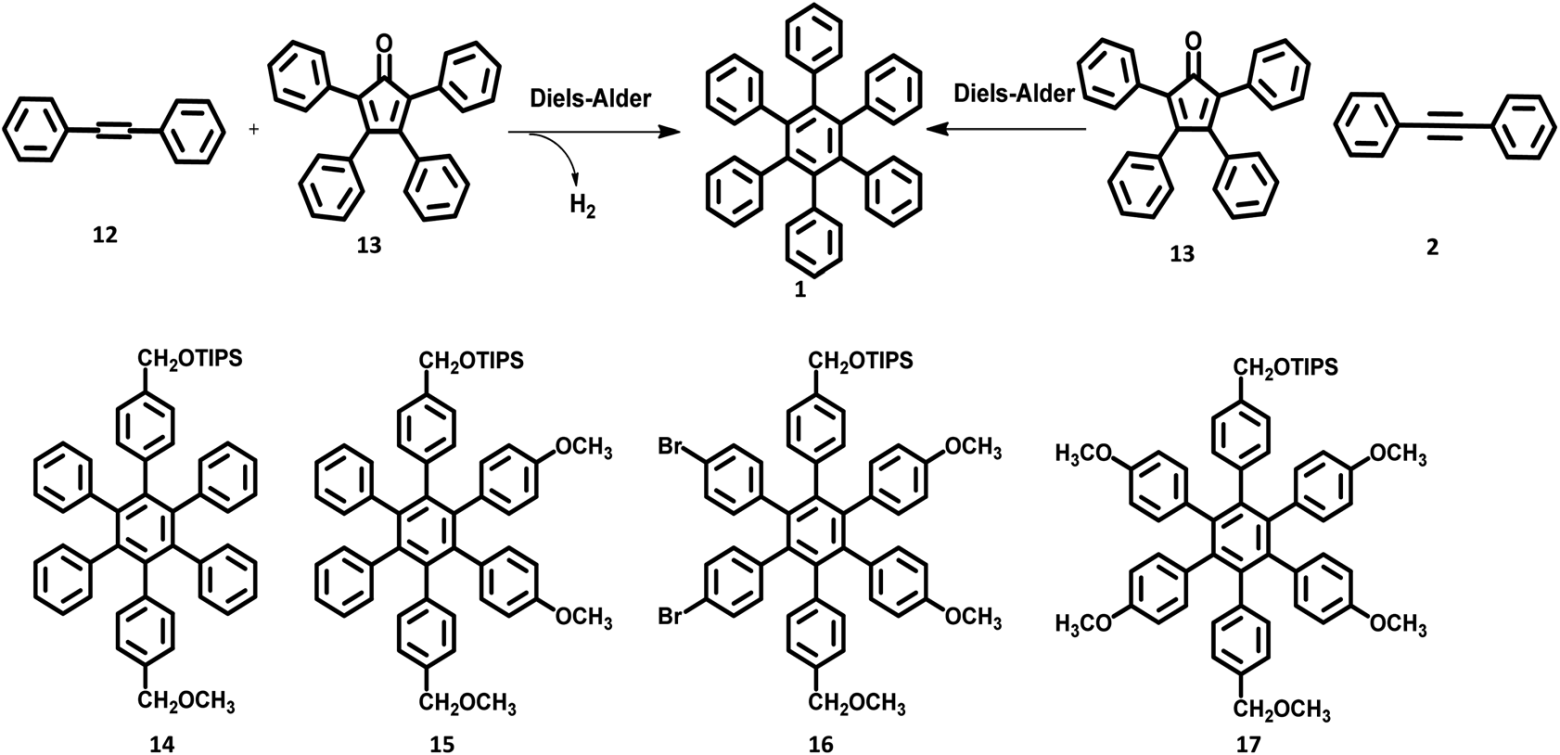
Synthetic scheme of Diels–Alder cycloaddition reaction.

Quite elegant and facile access to HAB derivatives is the Diels–Alder [4 + 2] cycloaddition of suitably substituted tetracyclones with diphenylacetylenes. This involves the formation of Diels–Alder adducts as the intermediate, which spontaneously extrude CO at 230 °C to yield the corresponding hexaphenylbenzene.^55^ Despite the complex synthetic strategy involved in this method, it opens many possibilities for the production of unsymmetrical HAB molecules possessing a broad spectrum of applications.^46,67,68^ The first successful synthesis of the hexaphenylbenzene molecule 1*via* the Diels–Alder reaction was performed in the early twentieth century by Dilthey and co-workers.^22^ The HPB molecule was synthesized by the reaction of tetraphenylcyclopentadienone 13 with stilbene 12 upon the elimination of carbon monoxide and hydrogen gases as side products. Additionally, the synthesis of tetraphenylbenzene was reported by the reaction of tetraphenylcyclopentadienone with a simple maleic anhydride. Later, the synthetic route for symmetric HPB molecules was modified by Dilthey *et al.*^1^ by the reaction of 13 (diene) with diphenylacetylene (dienophile) followed by the evolution of CO gas (Fig. 3). This was followed by a series of synthetic reports for the conventional Diels–Alder reaction under elevated temperatures.^69,70^ The unsymmetrical HAB derivatives can be synthesized by the reaction of suitably substituted tetracyclone with diarylacetylene. Potter and Hughes^71^ reported the synthesis of a series of unsymmetrical HPB molecules through asymmetric carbonylative reaction. The coupling reaction of different *para*-substituted benzyl halides in the presence of Collman's reagent results in unsymmetrically substituted 1,3-diarylacetones, which condenses with benzil to afford the desired unsymmetrical tetracyclone. The Diels–Alder reaction of tetracyclone with diarylacetylene leads to the formation of the desired unsymmetrical HPB derivatives 14–17 (Fig. 3).

### 2.3. Transition metal-catalyzed C–C coupling reactions

**Fig. 4:**
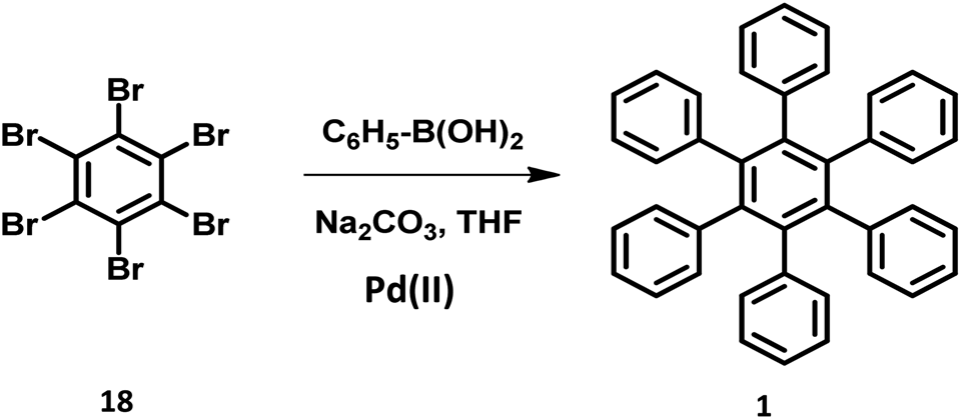
Schematic scheme of Suzuki reaction.

The synthesis of HPB molecules *via* the coupling reaction of hexabromobenzene with phenylmagnesium substrates were attempted by Durand in 1930. However, it resulted in the formation of tetrasubstituted 1,2,4,5-tetraphenylbenzene.^72^ Multiple Pd-catalyzed Suzuki coupling reactions were utilized for the synthesis of symmetric to differently substituted unsymmetrical HABs.^47,71,73–75^ The steric repulsions, separation difficulties, and less arylated byproducts in the reaction make the Suzuki cross-coupling more challenging than other traditional reactions. However, the sophisticated metal catalyst containing bulky and electron-rich phosphine ligands promote the formation of the desired compounds.^76–78^ The symmetric HPB derivatives synthesized by the reaction of hexabrominated or hexachlorinated benzene with phenylboronic acid using Pd(ii) catalyst are obtained in considerably good yields (Fig. 4).

### 2.4. Miscellaneous synthetic routes

**Fig. 5:**
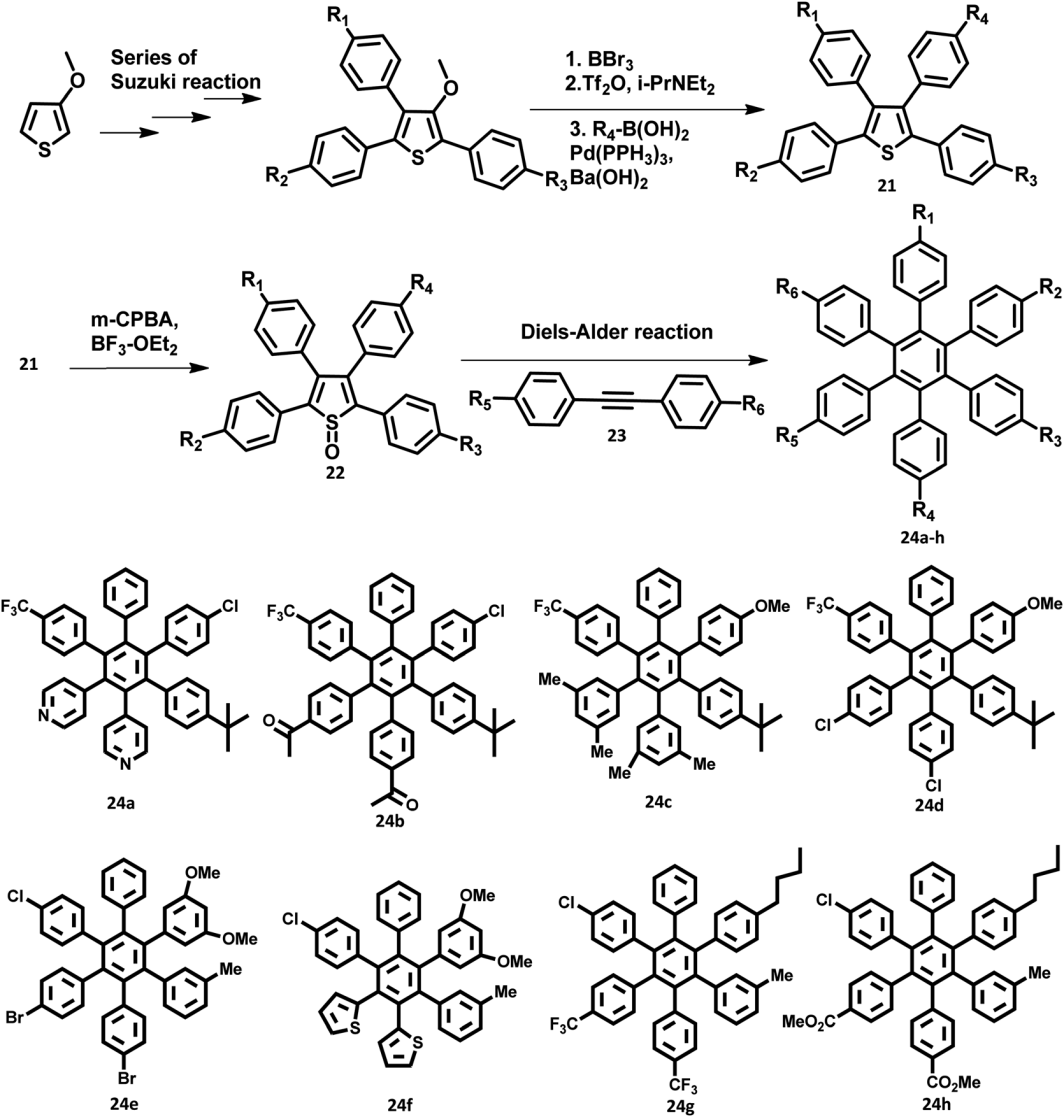
Synthetic scheme to prepare 24a–h.

**Fig. 6:**
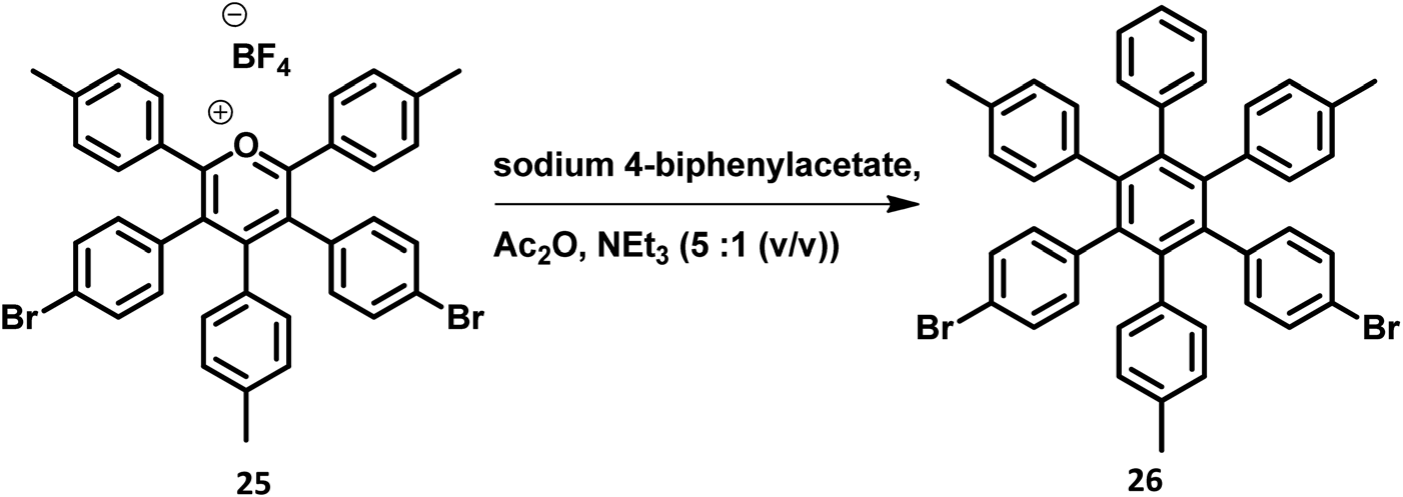
Synthetic scheme for the condensation reaction.

The development of synthetic strategies for the preparation of new HAB derivatives is a subject of continuing research. With a combination of multiple coupling reactions followed by Diels–Alder reaction, Suzuki and his research team^47^ synthesized HPB derivatives containing six different aryl groups. They have utilized the programmed synthetic sequence of C–H activation, Suzuki coupling, and Diels–Alder reaction to prepare unsymmetrical HAB derivatives (Fig. 5). The Diels–Alder precursor 22 was prepared by performing a series of multiple cross-coupling reactions followed by oxidation with *m*-CPBA. Finally, the precursor 24a was reacted with the corresponding diarylacetylene to yield unsymmetrical HAB derivatives 24a–h (Fig. 5) by [4 + 2] cycloaddition along with the evolution of sulfur oxide gas. Hiraoka *et al.*^79^ reported a condensation method to prepare 26 from arylacetates and pyrylium salts 25 obtained from the diastereomeric mixture of diketone. This approach enables the large-scale preparation of novel HPB derivatives that are difficult to produce under the traditional methods (Fig. 6).

## 3. Crystal structure of HPB

**Fig. 7:**
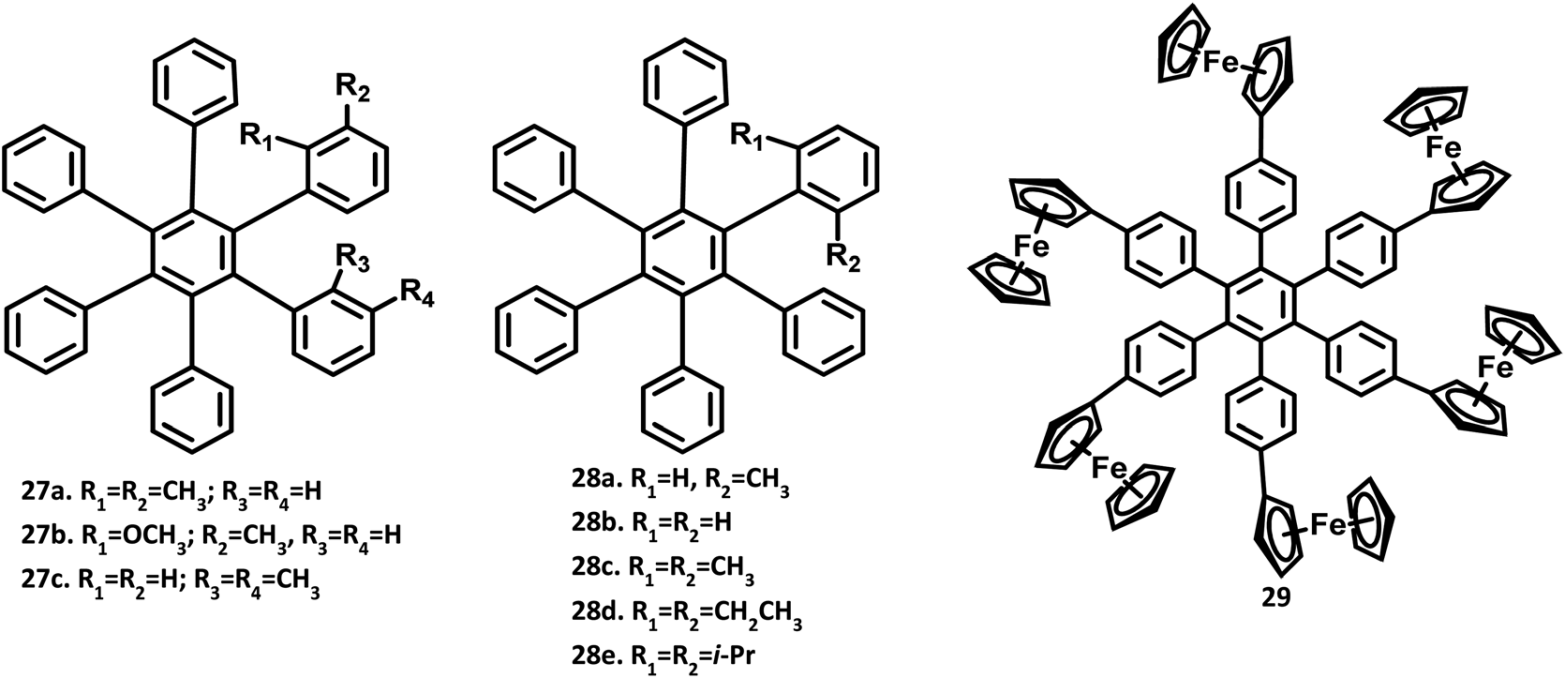
Molecular structure of HPB derivatives 27–29.

The structure of hexaphenylbenzene was determined by the single-crystal XRD technique in 1986 by Bart.^2^ There are two polymorphic forms for HPB, namely (i) pyramidal, and (ii) orthorhombic. According to the report, there is no significant deviation from the regular hexagonal structure observed for HPB. The HPB molecule adopts a nearly six-fold symmetry along with a propeller structure. The peripheral phenyl rings are not completely perpendicular to the horizontal central benzene ring but are twisted by about 25° from the central benzene. The bond angle and distance in the benzene rings are normal, with the C–C bridge distance in the range of 1.47 to 1.53 Å. The out of plane bending of peripheral ring bonds causes the high distortion of the molecule. This was followed by the X-ray crystal structure of HPB reported by Gust,^3^ which also confirms the perpendicular position of the peripheral rings to the central benzene ring. The molecular models suggest that the peripheral rings cannot lie in the horizontal axis due to steric hindrance. Furthermore, the *ortho*- or *meta*-substituted HPB molecules 27a–c exhibited stereoisomerism due to the restricted rotation about the single bond attached to the central benzene (Fig. 7). Besides, the peripheral rings are ∼65° perpendicular to the central benzene ring.

Wuest and team^4^ studied the cohesion in the crystals of *ortho*-substituted HPB molecules 28a–e. HPB and analogs show strong C–H⋯π interactions within the central benzene ring. The addition of substituents at the *ortho* position can block these interactions by changing the molecular cohesion. The *ortho* substituent showed systematic changes in the molecular packing, density, packing indices, melting point, the temperature of sublimation, and ratios of C⋯C, H⋯C, and H⋯H interactions.

Another interesting single-crystal form of symmetrical HPB derivative substituted with the ferrocene metal complex 28c was reported by Rathore and co-workers.^80^ The dihedral angle between the peripheral phenyl rings and central core benzene was observed in the range of 54.1 to 66.1°, as observed in other HPB derivatives. However, the dihedral angle and orientation of the ferroceneylcyclopentadienyl groups to the adjacent peripheral phenyl rings are much more varied in the range of 4.3 and 31.2°. This deviation of the phenylcyclopentadienyl coplanarity from the ideal aryl-ferrocene molecules could be attributed to the effective C–H⋯π interactions. Thus, there is variation in the dihedral angle with a change in the substituents due to the steric strains, resulting in the formation of different architectures.

## 4. Organic electronic applications of hexaaryl and pentaarylbenzene derivatives

### 4.1. Organic light-emitting diodes

OLEDs have received great attention due to their applications in solid-state lighting, wide-angle, and flat-panel displays.^81–87^ Particularly, electroluminescent molecules with extended π conjugations are potential candidates for hole injection and hole transport within the system.^88^ Most of the organic electroluminescent materials developed so far possessed high quantum yield (*Φ*) in solution but very low quantum efficiency in solid-state due to the presence of aggregates, exciplexes, excimers, and impurities within the film that can cause non-radioactive decay.^89–92^ But the amorphous films can efficiently suppress over-aggregation, which results in a stable emission and higher quantum efficiency.

One of the techniques used to fabricate amorphous films is the development of a molecule with a non-planar and asymmetric structure. Small molecules with hyper-branched structures or star-shaped dendrimers are considered to be ideal due to the suppressed intermolecular aggregation and well-defined energy transfer pathway within the molecule. Besides, the molecule possesses a 3D network that offers rigidity to afford high glass transition temperature along with an amorphous propensity within the films.^51,93^ Another interesting method incorporated to reduce the concentration effect is the doping approach, which is mainly used in highly efficient fluorescence and phosphorescence OLEDs.^94–99^ Most of the conjugated organic molecules were reported to emit three primary colored radiations, namely red, blue, and green emissions.^100–105^ Moreover, the primary color emitters are required to show a long lifetime, EL efficiency, and pure color coordinates to be applicable in large full-color displays. For instance, the blue emitters require a high intrinsic bandgap, although it hampers the balanced charge injection that further impairs the efficiency.^106^ Herein, the application of HAB-based simple molecules, dendrimers, and polymers in OLEDs has been discussed in detail. The detailed OLED parameters of all HAB derivatives are summarized in Table 1.

**Table 1:** OLED properties of HAB derivatives

	Properties of the emissive material			Device performance					Ref.
	HOMO/LUMO (eV)	*T*_g_/*T*_d_	*Φ*_PL_ in solution	Color	Active layer	CIE(*x*,*y*)	*η*_ext_ (%)	LE (cd m^−2^)	
30c	—	120/370	54	Blue	30c/TPBI	—	4.10	5650	107
31a	−5.10/−2.00	—/332	33^a^	Blue	PEDOT:PSS/31a:PVK /BCP/Alq_3_/LiF	0.188, 0.179	0.16	—	51
31b	−5.06/−1.81	124/296	14^a^	Blue	PEDOT:PSS/31b:PVK/BCP/Alq_3_/LiF	0.200, 0.186	0.15	—	51
31c	−4.90/2.06	100/306	35^a^	Blue	PEDOT:PSS/31c:PVK/BCP/Alq_3_/LiF	0.158, 0.201	1.6	1120^b^	51
31d	−4.96/1.71	118/326	7^a^	Blue	PEDOT:PSS/31d:PVK/BCP/Alq_3_/LiF	0.197, 0.201	0.14	—	51
32a	−5.92/−2.23	—/452	91	Blue	NCB/CPB/32a:FIrpic/BCP/Alq_3_	0.18, 0.37	2.61	5113^c^	109
32b	−5.94/−2.31	121/465	77	Blue	NCB/CPB/32b:FIrpic/BCP/Alq_3_	0.18, 0.37	2.08	3749^f^	109
32c	−5.73/−2.13	—/427	85	Blue	NCB/CPB/32c:FIrpic/BCP/Alq_3_	0.20, 0.38	4.00	4136^e^	109
32d	−5.83/−2.25	—/447	88	Blue	NCB/CPB/32d:FIrpic/BCP/Alq_3_	0.19, 0.42	5.59	5999^e^	109
33a	−5.76/−2.20	—/489	77	Blue	NCB/CPB/33a:FIrpic/BCP/Alq_3_	0.18, 0.34	3.49	2846^d^	109
33b	−5.83/−2.33	191/476	87	Blue	NCB/CPB/33b:FIrpic/BCP/Alq_3_	0.19, 0.44	6.20	4484^b^	109
34a	−6.20/—	—	72	Blue	TBPAH-PTPDES/34b/FIrpic-34a/TAZ/LiF	—	11	12 500	112
34b	−5.82/—	—	62	Blue	TBPAH-PTPDES/34b/FIrpic-34a/TAZ/LiF	—	11	12 500	112
35	−5.76/−3.60	/340	32, 92^g^	Red	NPB/35 (20 nm)/TPBI/LiF	—	0.43	835	89
					NPB/35 (50 nm)/TPBI/LiF		1.0	1572	
36	−6.20/−3.30	189/450	—	Orange-red	(i) NPB/36	0.63, 0.35	0.2	261	30
36	−6.20/−3.30	189/450	—	Green	(ii) NPB/CBP/36	0.32, 0.48	—	1840	30
				Blue	(iii-m) 100 nm: NPB/37/36	0.16, 0.26	—	3622	30
				Blue	(iii-n) 80 nm: NPB/37/36	—	—	3179	30
37	−5.70/−2.60	197/488	—	Blue	(iii-o) 60 nm: NPB/37/36	—	—	2123	30
				Blue	(iv-m) 100 nm NPB/CBP/37/36	0.16, 0.28	—	6230	30
				Blue	(iv-n) 80 nm NPB/CBP/37/36	—	—	5787	30
				Blue	(iv-o) 60 nm NPB/CBP/37/36	—	—	2932	30
38	—	405/464	36	Green	PEDOT:PSS/NPB:Alq_3_:38/TPBI/LiF	—	—	23458^h^	93
38	—	405/464	36	Green	PEDOT:PSS/38/TPBI/LiF	—	—	496^i^	93
39	−5.67/−3.26	—/421	∼1	Blue-violet	NPB/39/NPB	0.16, 0.05	3.98	—	114
40a	—	131/442	58.7	Blue	MoO_3_/NPB/40a/TPBI	0.16, 0.08	0.72	734	115
40b	—	—/473	34.3	Blue	MoO_3_/NPB/40b/TPBI			709	115
41a	−5.26/−2.09	128/454	68.6	Deep-blue	MoO_3_/NPB/41a/TPBI	0.15, 0.07	1.04	2090	115
41a	−5.26/−2.09	128/454	68.6	Deep-blue	MoO_3_/NPB (60 nm)/mCP/41a/TPBI/LiF	0.15, 0.08	2.30	3907	115
41a	−5.26/−2.09	128/454	68.6	Blue-violet	MoO_3_/NPB (40 nm)/mCP/41a/TPBI/LiF	0.15, 0.06	2.18	1498	115
41a	−5.26/−2.09	128/454	68.6	Deep-blue	MoO_3_/NPB/mCP/BmPyPb: 5% 41a/BmPyPb/TPBI/LiF	0.16, 0.10	1.44	3334	115
41a	−5.26/−2.09	128/454	68.6	Deep-blue	MoO_3_/NPB/mCP/BmPyPb: 10% 41a/BmPyPb/TPBI/LiF	0.16, 0.09	1.98	4121	115
41a	−5.26/−2.09	128/454	68.6	Deep-blue	MoO_3_/NPB/mCP/BmPyPb: 20% 41a/BmPyPb/TPBI/LiF	0.15, 0.09	2.35	5993	115
41a	−5.26/−2.09	128/454	68.6	Deep-blue	MoO_3_/NPB/mCP/BmPyPb: 30% 41a/BmPyPb/TPBI/LiF	0.16, 0.11	2.83	4653	115
41a	−5.26/−2.09	128/454	68.6	Deep-blue	MoO_3_/NPB/mCP/BmPyPb: 40% 41a/BmPyPb/TPBI/LiF	0.16, 0.11	3.85	5063	115
41a	−5.26/−2.09	128/454	68.6	Deep-blue	MoO_3_/NPB/mCP/BmPyPb: 50% 41a/BmPyPb/TPBI/LiF	0.16, 0.11	3.98	4746	115
41b	−5.63/−2.15	137/468	80.3	Blue	MoO_3_/NPB/41b/TPBI	0.15, 0.08	0.39	1088	115
41c	−5.26/−1.86	101/390	7.2	Blue	MoO_3_/NPB/41c/TPBI	0.15, 0.11	0.33	922	115
41d	−5.62/−2.08	113/445	6.4	Blue	MoO_3_/NPB/41d/TPBI	0.15, 0.09	0.22	826	115
41e	−5.60/−2.19	112/460	45.7	Blue	MoO_3_/NPB/41e/TPBI	0.15, 0.09	0.43	1105	115
42	−5.70/−2.97	292/426	—	Blue	2-TNATA/NPB/42/TPBI	0.20, 0.31	—	—	116
43	−5.43/2.68	340/467	—	Blue	2-TNATA/NPB/43/TPBI	0.20, 0.36	—	—	116
44	−5.54/2.49	340/449	—	Blue	2-TNATA/NPB/44/TPBI	0.15, 0.07	—	—	116
45	−5.7/−2.6	140/460	—	Blue	PPBI/45/mCBP: 15 wt% 4CzIPN/DBT-TRZ/DPB 20 wt% Liq/Libpp	—	8.5	—	117
45	−5.7/−2.6	140/460	—	Blue	PPBI/NPD/45/mCBP: 15 wt% 4CzIPN/DBT-TRZ/DPB: 20 wt% Liq nm)/Libpp	—	21.6	—	117
46a	−5.02/−2.68	—/450.4	5.5/61.5^j^	Bright-yellow	HATCN/TAPC/TCTA/46a/TmPyPB/LiF	0.39, 0.57	12.7	—	118
46b	−5.23/−2.79	—/464.9	9.1/51.8^j^		HATCN/TAPC/TCTA/46b/TmPyPB/LiF	0.28, 0.58	6.5	—	118
47	−5.28/−3.05	—/569	—	Green	47/Alq_3_	—	1.2–1.6	14 000–20 000	124
48a	—	80/379	—	Green	48a/Alq_3_	—	1.2–1.6	14 000–20 000	124
48b	—	111/463	—	Green	48b/Alq_3_	—	1.2–1.6	14 000–20 000	124
48c	—	96/399	—	Green	48c/Alq_3_	—	1.2–1.6	14 000–20 000	124
49b	—	—	608	Red-orange	PEDOT:PSS/49b	—	—	—	125
50a	—	—	610	Red-orange	PEDOT:PSS/50a	—	—	—	125
50b	—	—	608	Red-orange	PEDOT:PSS/50b	—	—	—	125
53	−5.46/−2.36	202/>500		Green	53/Alq_3_/LiF	—	—	22660^l^	127
54	—	—	14.8	Deep-blue	PEDOT:PSS/54/TPBI/CsF	0.17, 0.10	—	400^m^	128
55	−5.0/−2.6	—	39/8^k^	Green	PEDOT:PSS/55/TPBI/LiF	0.33, 0.66	0.3	3340	129
56	−5.0/−2.6	—	49/22^k^	Green	PEDOT:PSS/56/TPBI/LiF	0.31, 0.63	1.6	7030	129
57a	−5.0/−2.6	—	53/30^k^	Green	PEDOT:PSS/57a/TPBI/LiF	0.30, 0.63	1.7	5900	129
57b	−5.0/−2.6	—	56/36^k^	Green	PEDOT:PSS/57b/TPBI/LiF	0.29, 0.58	0.3	2040	129
55	−5.0/−2.6	—	39/8^k^	Green	PEDOT:PSS/55 (30 wt%)/TPBI/LiF	0.32, 0.62	1.0	—	129
56	−5.0/−2.6	—	49/22^k^	Green	PEDOT:PSS/56 (30 wt%)/TPBI/LiF	0.31, 0.63	4.4		129
57a	−5.0/−2.6	—	53/30^k^	Green	PEDOT:PSS/57a (30 wt%)/TPBI/LiF	0.30, 0.63	6.1	—	129
57b	−5.0/−2.6	—	56/36^k^	Green	PEDOT:PSS/57b (30 wt%)/TPBI/LiF	0.29,0.58	0.6		129
58	—	—	88	Blue	ITO/PEDOT:PSS/58/TPBI/CsF	0.15, 0.18	—	728	120
59	—	—	73	Blue	ITO/PEDOT:PSS/59/TPBI/CsF	0.15, 0.17	—	1440	120
60	−5.06/−2.42	—	6	Blue	PEDOT:PSS/(TCCz):60	0.72, 0.92	—	—	119
61a	−5.06/−2.42	—	7	Red	PEDOT:PSS/(TCCz):61a	0.72, 0.91	—	—	119
61b	−5.06/−2.42	—	9	Red	PEDOT:PSS/(TCCz):61b	0.72, 0.91	—	—	119
63	−5.65	91/433	82/74	Blue	PEDOT:PSS/63	0.17, 0.08	6.1	998	130
64	−5.56	103/434	82/82	Blue	PEDOT:PSS/64	0.16, 0.08	6.7	1707	130
65	−5.51	114/434	84/92	Blue	PEDOT:PSS/65	0.16, 0.07	6.8	1962	130
66	−5.10/−2.14	123/430	77	Blue	PEDOT:PSS_4083_/66/TPBI/LiF	0.149, 0.104	—	9524	106
66	−5.10/−2.14	123/430	77	Blue	PEDOT:PSS_8000_/66/TPBI/LiF	0.154, 0.136	—	6378	106
67	−5.10/−2.15	142/429	88	Blue	PEDOT:PSS_4083_/67/TPBI/LiF	0.151, 0.136	—	8596	106
67	−5.10/−2.15	142/429	88	Blue	PEDOT:PSS_8000_/67/TPBI/LiF	0.154, 0.150	—	6057	106
68a	−5.36/−2.40	—	0.65^n^/0.71^o^	Blue	PEDOT:PSS/68a	0.16, 0.15	—	112	131
68a	−5.36/−2.40	—	0.65^n^/0.71^o^	Blue	PEDOT:PSS/TFB/68a/PEGPF/Cs_2_CO_3_	0.16, 0.19	—	1450	131
68b	−5.59/−2.44	—	0.59^n^/0.75^o^	Blue	PEDOT:PSS/68b	0.16, 0.16	—	395	131
68b	−5.59/−2.44	—	0.59^n^/0.75	Blue	PEDOT:PSS/TFB/68b/PEGPF/Cs_2_CO_3_	0.17, 0.20	—	1526	131
68c	−5.27/−2.35	—	0.59^n^/0.53^o^	Blue	PEDOT:PSS/68c	0.16, 0.20	—	715	131
68c	−5.27/−2.35	—	0.59^n^/0.53^o^	Blue	PEDOT:PSS/TFB/68c/PEGPF/Cs_2_CO_3_	0.16, 0.21	—	3726	131
68c	−5.27/−2.35	—	0.59^n^/0.53^o^	Blue	PEDOT:PSS/TFB/68c	0.16, 0.20	—	—	131
68d	−5.50/−2.46	—	0.39^n^/0.70^o^	Blue	PEDOT:PSS/68d	0.19, 0.28	—	195	131
70	−5.52/−2.45	—	—	Blue	PEDOT:PSS/70/Ca/Al	0.16,0.10	—	—	132
71	—	—	—	—	PEDOT:PSS/71/TSPO1/TmPyPB/LiF/Al	0.26, 0.48	3.1	7860	133
72	—	—	—	—	PEDOT:PSS/72/TSPO1/TmPyPB/LiF/Al	0.30, 0.54	3.5	6910	133

a *η*_f_ values.

b At 14 V.

c At 15 V.

d At 15.5 V.

e At 16 V.

f At 17 V.

g At pH > 8.

h At 14 V.

i At 15.7 V.

j Taken in the film.

k Taken in film.

l At 12 V.

m At 10 V.

n Excited w.r.t surface.

o Excited w.r.t core.

#### 4.1.1 Simple and linear analogues of hexaarylbenzene

**Fig. 8:**
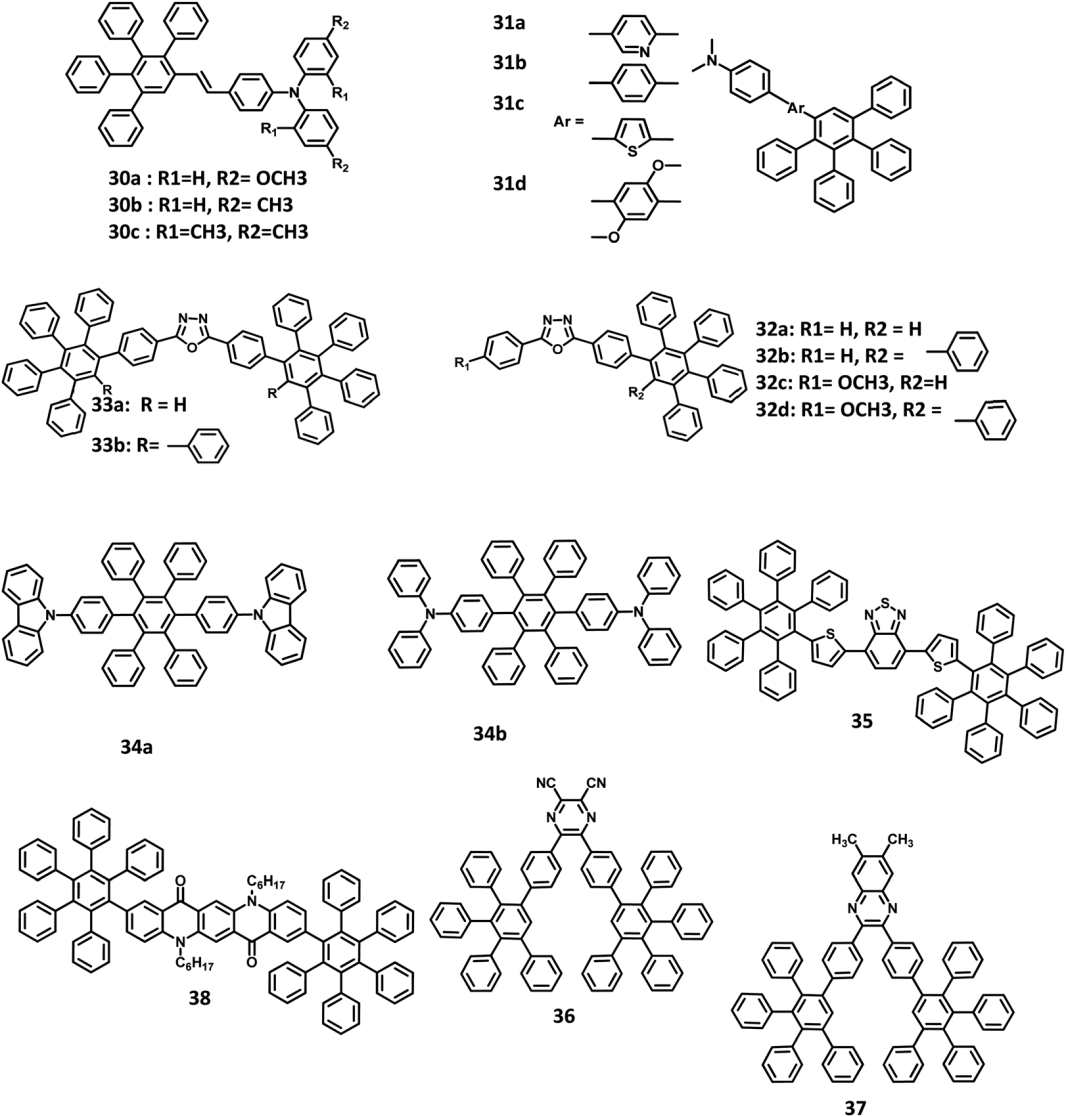
Molecular structure of HAB derivatives 30–37.

Simple polyarylphenyl (PAP) derivative-appended starburst units feature efficient conjugation and weak interaction towards molecular aggregation in the solid-state, as they behave as excellent chromophores for application in EL devices. Besides, the combination of PAP derivatives with bulky alkyl segments, such as *tert*-butyl, tetraphenylsilyl, and isopropyl units, prohibits molecular aggregation. The PAP units within the non-planar triarylamine molecules possessing alkyl units promote the formation of molecular glasses and improvement of fluorescence in the solution as well as solid-states. Chen *et al.*^107^ reported a new series of PAB-attached *ortho*-substituted arylamine molecules for application in efficient blue emissive non-doped OLEDs (Fig. 8). These derivatives 30a–c possess good thermal stability, stable glass transition (*T*_g_ = 110–120 °C), and decomposition temperatures (*T*_d_ = 348–370 °C). The absorption maxima values of the derivatives are observed in the range of 370–382 nm. Compared to other derivatives, the 30c molecule showed a relatively high quantum yield (*Φ*_f_ = 54%) with a deep blue-color hue either in the solid film (*λ*maxem = 465 nm) or solution state (*λ*maxem = 462 nm). The 30c molecule is observed to have intense and highly efficient blue emission, which is attributed to the presence of fluorophores in the *ortho* position of triphenylamine. Thus, the non-doped OLED fabricated as ITO/30c (60 nm)/TPBI (40 nm)/Mg:Ag, where, (1,3,5-tris(*N*-phenylbenzimidazol-2-yl)benzene), is abbreviated as TPBI.^108^ The device showed a predominant EL band at 462 nm. This is similar to the solid-state PL spectrum (30c), which reveals that the emission originates from the fluorophore. Furthermore, the PAB-arylamine-based OLED device showed luminance at around 5650 cd cm^−2^, with a driving voltage of 6 V, and external quantum efficiency (EQE) of 4.1% cm^2^. Besides, the deep blue emissions were observed in the reported devices with the EL values in the range of 640–940 cd m^−2^ and a current density of 20 mA cm^−2^. Thus, the strongly suppressed aggregation behavior makes the PAP segments ideal for EL and energy harvesting devices. The interesting molecular geometry of the HAB molecule intrigued many researchers to develop new type of OLED devices with improved performance.^26^

Chen and co-workers^51^ reported the development of novel solution-processable blue light-emitting materials consisting of blue donor–acceptor (D–A) systems bearing PAB-attached arylamine derivatives (Fig. 8). The molecules are substituted with different conjugation bridges connected to the electron-donating *N*,*N*-dimethylaminophenyl moiety at one end and tetraphenylphenyl group at the other. The *N*,*N*-dimethylaminophenyl group increases the HOMO energy level, which regulates hole injection/transport and thus enhances the device efficiency. The electron-donating 2,5-dimethoxyphenyl, phenyl, and thienyl moieties and electron-withdrawing pyridyl rings are used as the central bridges to tune the charge-transporting properties of the molecules. The bandgap energy of the 31a–d derivatives was determined to be 3.10, 3.25, 2.84, and 3.25 eV, respectively. It is seen that the HOMO–LUMO energy gap of the compounds is tunable by varying the bridge between the alkyl amine and HPB moieties. Among the derived molecules, 31c possessed the highest HOMO and lowest LUMO level, along with the lowest energy gap due to the strong electron-donating nature of the thienyl aromatic ring. Thus, the multilayer devices were fabricated with the four molecules and configuration of ITO/PEDOT:PSS (50 nm)/31a or 31b or 31c or 31d:PVK (2%, wt/wt) (50 nm)/BCP (10 nm)/Alq_3_ (20 nm)/LiF (0.5 nm)/Al (200 nm), where PVK represents poly(*N*-vinylcarbazole) and BCP is 2,9-dimethyl-4,7-diphenyl-1,10-phenanthroline. Among the molecules, 31c exhibited high-quality blue emission with the CIE coordinates of (0.158, 0.201), while the other molecules emitted sky blue color. The derivative 31a showed a *V*_on_ value of 8 V, *L*_max_ of 1120 cd m^−2^, and the maximum brightness of 1.0 cd m^−2^. The emission wavelength, current/quantum efficiencies, and CIE coordinates of the devices are almost similar for all other molecules. The EQE and current efficiency of 31c were determined to be 1.6% and 2.2 cd A^−1^, respectively. However, the other three derivatives showed low efficiencies (EQE = ∼0.15% and current efficiency = ∼0.15 cd A^−1^). This can be attributed to the mismatch in the energy levels between 31b and 31d compounds incorporated with the host material (PVK). Thus, the high device performance of 31c is attributed to the better hole/electron injection and transport characteristics of PVK.

An alternative way to develop efficient OLED behavior along with color purity is by the introduction of a dopant emitter to the host material. However, it is difficult to discover a suitable electrophosphorescent host material for blue EL devices, owing to their high triplet energy level that does not favor transport. Liu and co-workers^109^ employed HAB/HPB-attached oxadizole derivatives 32a–d, and 33a, b as efficient host materials for application in blue electrophosphorescent organic light-emitting diodes (PhOLED) (Fig. 8). All the compounds exhibited excellent amorphous propensity and good thermal stability. Besides, the π–π stacking was absent in the film of the 32a model compound, which is reflected in its optical properties. The absorption spectra exhibited a band at the lower energy region (*λ*_max_ = 320 nm), which originated from the π–π* transition of the oxadiazole skeleton, and another band at the higher energy region (*λ*_max_ = 240 nm) due to the π–π* transition of the phenyl rings. Furthermore, a significant increase in the molar extinction coefficient at the higher energy region reflects the increase in the number of phenyl units within the compound. The *Φ*_PL_ of the 32a–d and 33a, b derivatives were measured to be in the range of 77–91%. The band gap values of the materials were high, which promote their application in blue, green, or red light-emitting devices. Thus, the devices were fabricated with the 32a–d and 33a, b derivatives, along with a dopant in the configuration of ITO/NPB (20 nm)/CBP (20 nm)/32a or 32b or 32c or 32d or 33a or 33b:FIrpic (40 nm)/BCP (20 nm)/Alq_3_ (5 nm)/LiF (1 nm)/Al (100 nm), where, Alq_3_, NPB,^110^ BCP, and CBP are the ETL, HTL, HBL, and additional HTL material, respectively. Iridium(iii)bis[4,6-difluorophenylpyridinato-N,C29]picolinate (FIrpic)^111^ and LiF/Al are the corresponding host emitter and cathode used in the device. The device containing 33b showed the best performance with a maximum EQE of 6.20% and *L*_max_ of 4484 cd m^−2^. This is attributed to the low lying LUMO level of 33b, which facilitates electron injection by reducing the energy barrier.

Due to the effective emission behavior of the triplet and singlet excitons, the internaxl quantum efficiency of the electrophosporescent OLEDs reached 100%. Watanabe and Junji^112^ reported two novel HPB derivatives with high triplet excited energy (T1) levels for application in blue PhOLEDs (Fig. 8). All the derivatives were synthesized using the Diels–Alder cycloaddition reaction. Due to the non-planar structure of the HPB units, the extensive conjugation of 34a and 34b derivatives was reduced compared to that of the conventional carrier transport and host materials such as *N*,*N*′-dicarbazolyl-4,4′-biphenyl (CBP). The photoluminescence quantum efficiencies (PLQE) of 34a and 34b were calculated to be 72 and 62%, respectively. Hence, the blue OLED devices were fabricated with FIrpic as the EML, 34a and 34b derivatives as the carrier transport layers. Also, the buffer layer of (poly(aryleneethersulfone)-appended tetraphenylbendizine (PTPDES)-doped tris(4-bromophenyl)aminium hexachloroantimonate^113^ (TBPAH)) layer was introduced at the anode. Accordingly, the device was fabricated with the configuration of ITO/TBPAH (10 wt%)-doped PTPDES (20 nm)/34b (30 nm)/FIrpic (8 wt%)-doped 34a (30 nm)/TAZ (30 nm)/LiF (0.5 nm)/Al (100 nm), where, TAZ is 3-(40-*tert*-butylphenyl)-4-phenyl-5-(400-biphenyl)-1,2,4-triazole. The EQE, power efficiency, and maximum luminescence were observed to be 11%, 12 lm W^−1^, and 12 500 cd m^−2^, respectively. Furthermore, a device fabricated with classic materials and a configuration of ITO/TBPAH (10 wt%)-doped PTPDES (20 nm)/NPD (30 nm)/FIrpic (8 wt%)-doped CBP (30 nm)/TAZ (30 nm)/LiF (0.5 nm)/Al (100 nm) was used to compare the performance of the HAB derivatives. An external quantum efficiency of 4.8% was observed for the device at 100 cd m^−2^. The result suggests that the high triplet excited energy levels of 34a and 34b are confined to the FIrpic compound, while the classic materials (NPD and CBP) act as the triplet quenchers due to their low T1 values.

Liu and team,^89^ further broadened the research by reporting HPB-attached benzo[2,1,3]thiadiazole derivative for application in red light-emitting non-doped OLEDs (Fig. 8). The corresponding HOMO and LUMO of 35 were determined to be around −3.60 eV, and −5.76 eV, respectively, which facilitates easy electron injection and hole transport mechanism. The emission spectra were observed at 609 nm, and *Φ*_PL_ of 35 was measured to be 32% in the solution and 92% at pH > 8.3. This weak excited-state solvatochromism indicates that the 35 derivative possesses minimal intermolecular π–π stacking and weak dipole–dipole interactions. Thus, the devices were fabricated with the configuration of ITO/NPB (30 nm)/35 (*x* nm)/TPBI (30 nm)/LiF (1 nm)/Al. Here, NPB, LiF, 35, and TPBI were used as HTL, EIL, EML, and ETL, respectively. The thicknesses of 35 varied from 20 to 50 nm to enhance the color purity of the device. The EL spectrum (609 nm) was similar to the emission spectrum of 35 for the thickness of 20 nm. While the thickness increased to 50 nm, the EL peak shifted to 621 nm with enhanced color quality. The other EL properties of devices were also improved with an increase in the emissive layer thickness. For an emissive layer thickness of 20 nm, the non-doped OLED acquired a brightness of 835 cd m^−2^ at 200 mA cm^−2^ and an EQE of 0.43%. When the thickness of 35 was increased to 50 nm, the performance of the device was improved with a current density of 200 mA cm^−2^, brightness of 1572 cd m^−2^, and a maximum EQE of 1.0%. Thus, the device performance was doubled without any concentration quenching upon increasing the EML thickness.

Many efficient red light-emitting materials were explored for OLEDs, although the development of blue-emitting materials with desirable properties remains a challenge. This is due to the low electron affinity (EA) and high energy gap of these materials that cause inefficient charge injection and transport. Thus, it is important to introduce/synthesize emitters with electron-withdrawing or accepting groups, such as cyanide, nitro, and fluorine, to increase the EA of the materials. Yu and co-workers^30^ reported a highly efficient blue OLED fabricated with novel electron-accepting 2,3-dicyano-5,6-di-(4-(2,3,4,5-tetraphenylphenyl)phenyl)pyrazine (36) and 6,7-dimethyl-2,3-di-(4-(2,3,4,5-tetraphenylphenyl)phenyl)quinoxaline (37) compounds (Fig. 8). Here, the derivative 36 was used as the emissive layer and 37 was synthesized to operate as an exciplex-eliminating OLED layer. To investigate the EL properties of the compounds, the devices were fabricated with the following configurations: (i) ITO/NPB (20 nm)/CBP (20 nm)/36 (40 nm)/Al (100 nm) and (ii) ITO/NPB (40 nm)/36 (40 nm)/Al (100 nm), where, NPB and CBP are the hole-transport materials. Here, 36 was used as an emissive as well as an electron-transporting layer. In device (ii), a second HTL of CBP (20 nm) was inserted between the 36 and NPB layers to increase the hole injection from the NPB to the CBP layer. Device (i) emitted a yellowish-green light, while the device (ii) exhibited reddish-orange emission along with a red-shifted wavelength, as seen from the PL spectra of 36 and 37. This behavior suggests that the emissions from devices (i) and (ii) originate from the exciplex formation of 36 on employing NPB and CBP as the electron-donating layers. To prevent exciplex formation, an additional layer possessing specific properties was inserted between the HTL (donor) and emissive layer (acceptor). The compound 37 was used as the exciplex-eliminating layer to prevent exciplex formation and acquire efficient blue-light emission. Further, the devices were fabricated with the configuration of (iii) ITO/NPB (40 nm)/37 (10 nm)/36 (50 nm)/Al (100 nm) and (iv) ITO/NPB (20 nm)/CBP (20 nm)/37 (10 nm)/36 (50 nm)/Al (100 nm). The device (iv) with an exciplex-eliminating layer exhibited the highest current efficiency of 5.2 cd A^−1^ and a maximum brightness of 6230 cd m^−2^. To analyze the influence of film thickness on the luminous efficiency and operating voltage, devices (iii) and (iv) were fabricated with organic layers of different film thicknesses. The turn-on and operating voltages were reduced, and the luminous efficiency was slightly changed for devices (iii) and (iv), while the thickness of the organic layers was reduced from 100 to 80 nm. The operating and turn-on voltages were further reduced, and the thickness of the organic films was decreased to 60 nm. The devices employed with the exciplex-eliminating layer exhibited better performance than those without the 37 layer. Thus, the electron-deficient cyano and pyrazine groups in the blue-OLED perform well as an electron-transport layer as well as the emissive layer.

A suppressed aggregation and solid-state emission were achieved in OLEDs by the synthesis of pentaphenylphenyl substituted quinacridone derivatives by Wang and team.^93^ Here, they have employed two PAB groups attached at the two terminals of quinacridone (Fig. 8). Compared to other quinacridone derivatives, compound 38 showed relatively higher emission in the solid film. Based on the desired properties, the OLED device was fabricated with a configuration of ITO/PEDOT:PSS (40 nm)/NPB:Alq_3_:38 (1–5 wt%) (40 nm)/TPBI (35 nm)/LiF (1 nm)/Al. The Alq_3_ mixture was used as the EML, while TPBI and PEDOT:PSS were used as HBL/ETL and HIL, respectively. On doping the NPB:Alq_3_ EML with 3% concentration of 38, the device was found to have the best performance, along with the radiation of green emission. The device showed maximum current efficiency of 10.0 cd A^−1^, *V*_on_ of 3.6 V, and *L*_max_ of 23 458 cd m^−2^. The molecule was also used to fabricate non-doped OLED with a configuration of ITO/PEDOT:PSS (40 nm)/38 (40 nm)/TPBI (35 nm)/LiF (1 nm)/Al, but the device was found to have poor performance. This device was found to have the highest efficiency of 0.07 lm W^−1^ and maximum luminance brightness of 496 cd m^−2^ at 13 V, which can be assigned to the decreased electron and hole mobilities (*μ*_e_ = 2.1 × 10^−7^ cm^2^ V^−1^ s^−1^ and *μ*_h_ = 5.3 × 10^−6^ cm^2^ V^−1^ s^−1^).

**Fig. 9:**
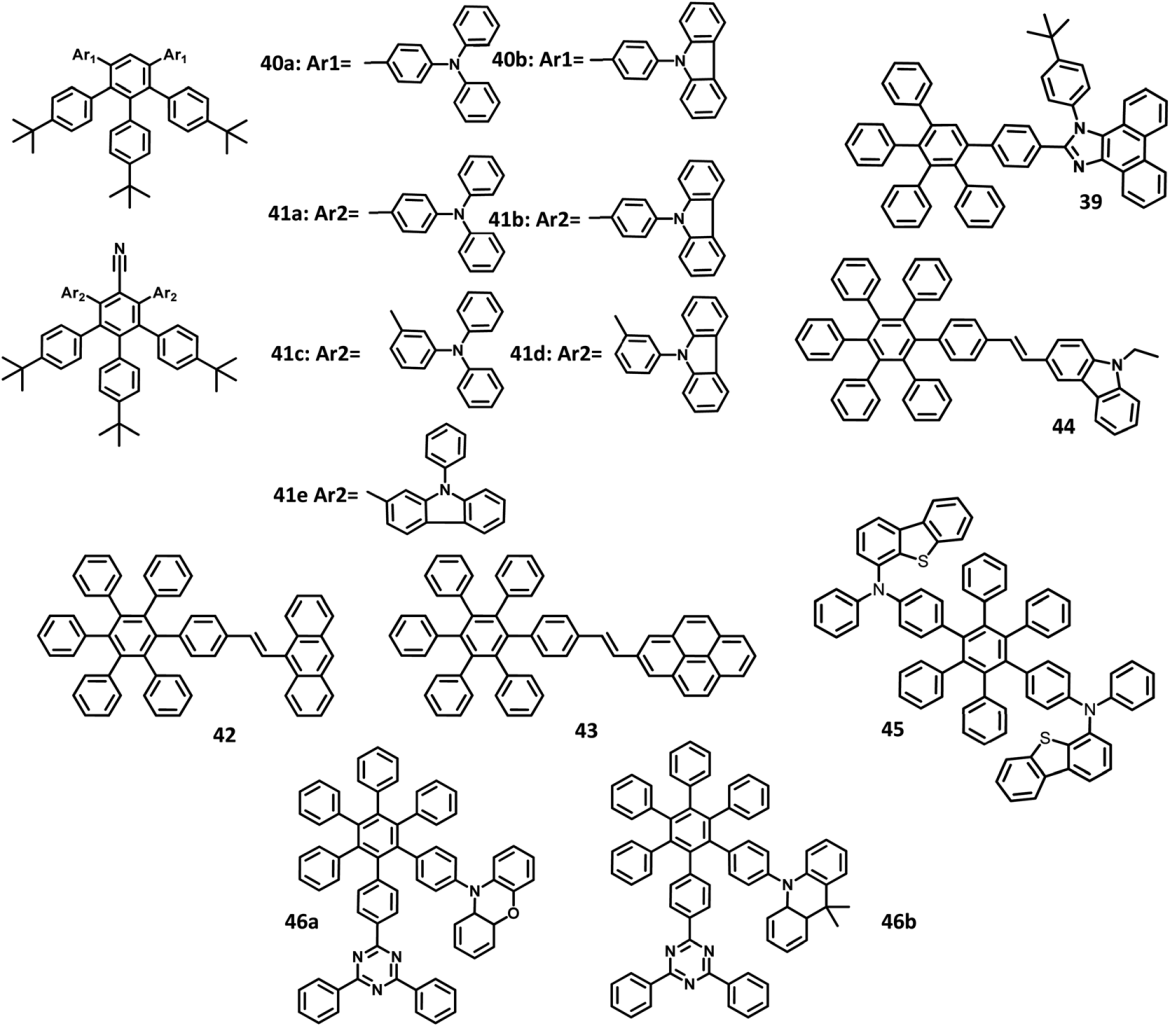
Molecular structure of HAB derivative 39–46.

Though many electrophosphorescent materials for EL applications have been reported in the literature, it is challenging to synthesize a molecule with balanced hole–electron charge transports. By the introduction of electron-donor and acceptor segments, it is possible to achieve a balanced charge transport with a broadened exciton region. Consequently, the device performance can be increased along with a decrease in the efficiency roll-off. In this demand, Lee and co-workers^114^ published a work on the synthesis of HAB-appended bipolar phenanthroimidazole derivatives for application in high-efficiency non-doped blue OLEDs (Fig. 9). The electron-donating and accepting groups present in the molecule induce bipolar transporting nature within the material. Furthermore, the fluorescence quantum yield of 39 was estimated to be almost 1 in the solution state, along with a bandgap value of 3.26 eV. Thus, the devices were fabricated with the bipolar compound 39 in ITO/NPB, (20 nm)/39 (80 nm)/NPB (20 nm)/Al (150 nm) and ITO/TPBI (20 nm)/39 (80 nm)/TPBI (20 nm)/LiF (1 nm)/Al (150 nm) for the development of hole-transport and electron-transport only devices, respectively. TPBI and NPB were used to inhibit the hole and electron injections from the anode and cathode, respectively. Here, TPBI was used as the ETL, TCTA was used as a buffer layer as well as a hole blocking layer. The electron and hole recombination was inhibited in the EML, which may be attributed to the large bandgap of TPBI (3.5 eV) and TCTA (3.4 eV). The compound exhibited blue-violet colored emission with the corresponding CIE coordinates of (0.16, 0.05), which is close to the standard value for blue emission (0.14, 0.08) following the NTSC standards. The hole injection barrier of NPB/TCTA and electron injection barrier of TPBI/EML is small, which enable the transport of electron and hole to the EML. Also, the device showed high *η*_ext_, *η*_c_, and *η*_p_ values of 5.02%, 2.10 cd A^−1^, and 1.88 lm W^−1^, respectively.

Due to π–π stacking or molecular aggregation, the emission spectra of the emitters show a redshift to the lower energy. Consequently, the emission moved from blue to the green-blue region. This can be resolved by the introduction of alkyl or bulky twisted substituents into the molecules. Later, Li and co-workers^115^ reported alkylated HPB-attached triphenylamine derivatives with high EQE values for application in deep-blue emission OLEDs. Besides, the HPB was substituted with nitrile groups to develop D–π–A type fluorophores. Moreover, a set of molecules were developed using the Suzuki coupling reaction, which is an alternative to the Diels–Alder reaction. Owing to rigid conformation, the *para*-linked 40a–41b derivatives show better thermal stability than *meta*-linkage derivatives (Fig. 9). The emission spectra of all the compounds were observed at 400–450 nm except for 40b (367 nm). The compounds 41a and 41b exhibited the highest *Φ* value in the solution (68.6 and 80.3%).

Some compounds showed higher HOMO than NPB (−5.30 eV), which contribute to better charge transfer. To examine the EL performance of the derivatives, seven non-doped OLEDs were fabricated with the configuration of ITO/MoO_3_ (10 nm)/NPB (60 nm)/40a or 40b or 41a or 41b or 41c or 41d or 41e (30 nm)/TPBI (30 nm)/LiF (1 nm)/Al, where, TPBI, MoO_3_, and NPB were used as HBL, HIL, and HTL, respectively. All the compounds exhibited deep blue emission in the EL spectra. The devices having D–π–A molecular system showed low *V*_on_ values compared to others, as a result of the reduced injection barriers between HTL and EML. Also, the devices incorporated with *meta*-linkage emitters possess non-extended conjugation to yield highly twisted structures and high *V*_on_ values. Among the fabricated devices, the one with compound 41a emitter was observed to have a maximum EQE of 1.04% and CIE coordinates of (0.15, 0.07), along with a deep blue colored emission. Based on 41a, the device architecture was further modified. An additive (mCP) was introduced between the EMLand NPB within the device with a configuration of ITO/MoO_3_ (10 nm)/NPB (60 nm)/mCP (10 nm)/10 (30 nm)/TPBI (30 nm)/LiF (1 nm)/Al, wherein, the mCP acts as a blocking layer to confine the excitons in the EML. Likewise, the device configuration was optimized with different layers and dopants for the device fabricated with compound 41a. The doped 41a-based devices were found to have a much-improved performance with a current efficiency of 4.51 cd A^−1^ and EQE of 3.98%. The color quality and EL characteristics suggest that the compound 41a is appropriate for deep-blue light-emitting applications.

The development of blue emitters with high efficiency, lifetime, and color purity is challenging. Park *et al.*^116^ reported a series of HPB core tailored with anthracene, pyrene, and carbazole segments for application in efficient electroluminescent devices. The structure–property relationship was also analyzed. The absorption spectra of the derivatives were observed at 390 nm for 42, 335 nm for 43, and 385 nm for 44 (Fig. 9). These derivatives exhibited emission maxima at 483, 453, and 488 nm for 42, 43, and 44, respectively. Furthermore, the emission of 43 was observed in the deep blue region, while other derivatives were shown to exhibit greenish-blue emission due to the concentrated electron density on the vinyl group that increased the conjugation of the compounds. The corresponding HOMO and LUMO levels of 43 were 5.54 and 2.49 eV, respectively, while those of 44 were −5.43, and 2.68 eV, respectively. Based on the above-trusted results, the OLED device was fabricated with the configuration of ITO/2-TNATA (60 nm)/NPB (15 nm)/42 or 43 or 44 (35 nm)/TPBI (20 nm)/LiF (1 nm)/Al (200 nm), in which, 2-TNATA (4,4′,4′′-tris(*N*-(2-naphthyl)-*N*-phenyl-amino)-triphenylamine) was served as the HIL. Among the derivatives, 44 had a higher current efficiency of 7.91 cd A^−1^ than the other two compounds (42 = 0.38 cd A^−1^ and 43 = 1.76 cd A^−1^). Moreover, 44 showed a low operating voltage, which is attributed to the higher HOMO and lower band gap values that further reduce the energy barrier between the ETL and HTL. The CIE values of 44, 42, and 43 showed emission in the deep blue and sky-blue region.

Sasabe and co-workers^117^ reported an HPB tailored dibenzothiophene molecule with good hole transport properties for blue OLEDs with thermally activated delayed fluorescence (TADF) behavior (Fig. 9). The compound 45 showed high *T*_g_ and *T*_d_ values over 140 °C and 460 °C, respectively. To evaluate the property of HTL, the OLED device was fabricated with the configuration of (i) ITO (100 nm)/PPBI (20 nm)/45 (20 nm)/mCBP: 15 wt% 4CzIPN (30 nm)/DBT-TRZ (10 nm)/DPB20 wt% Liq (40 nm)/Libpp (1 nm)/Al, where Libpp is 2-(2′,2′′-bipyridine-6′-yl)phenolate, where, PPBI represents 4-isopropyl-4-methyldiphenyl-iodonium tetrakis(pentafluorophenyl)borate and mCBP represents 3,3-di(9*H*-carbazol-9-yl)biphenyl. Also, DPB serves as the ETL, while DBT-TRZ serves as the HBL to attain prolonged operation stability. The EL device exhibited a low operating voltage of 2.7 V, leading to the formation of low-power consumption OLEDs, which is lower than the previously reported stable TADF devices. To improve the hole injection, an additional HTL (NPD) was introduced between the HTL and polymer buffer layer. Thus, the device structure was ITO (100 nm)/PPBI (20 nm)/NPD (10 nm)/45 (10 nm)/mCBP: 15 wt% 4CzIPN (30 nm)/DBT-TRZ (10 nm)/DPB: 20 wt% Liq (40 nm)/Libpp (1 nm)/Al. The device with additional HTL exhibited better performance of low *V*_1000_ and high *η*_ext,1000_ of 21.1% than single HTL layered device. Moreover, the device exhibited a longer operation lifetime at 90% of the initial luminance (LT_90_ = 313 h), along with an LT_50_value of 1000 h. The longer lifetime is attributed to the reduction of negative CT interaction and prevention of triplet exciton quenching as a result of the higher *E*_T_ of 4CzIPN. Thus, the molecule with TADF exhibited good OLED properties, which can be used in commercial applications.

Very recently, Zhao and team^118^ reported new “aggregation-induced delayed fluorescence” (AIDF) fluorophores comprising of acridine and phenoxazine as the donors and triazine as the acceptor, along with CT process for non-doped OLEDs (Fig. 9). The single crystal of 46b reveals that the six phenyl groups of HPB are in a twisted nature and are aligned along with short distances (<3 Å), indicating the strong electronic interactions of these phenyl groups *via* “through-space conjugation effect”. The absorption and emission for 46a were observed at 310 nm and 541 nm, respectively, while those of 46b were observed at 310 nm and 595 nm, respectively. Although the corresponding *Φ*_Sol_ values were very low (5.5–9.1%) in the solid-state, the compounds 46a, b showed higher *Φ*_film_ values of 51.8 and 61.5%, respectively. Besides, 46a, b possesses short mean lifetimes of 7.5 ns and 15.2 ns, respectively in the solution state. To investigate their EL properties, the OLED device was fabricated with a configuration of ITO/HATCN (5 nm)/TAPC (20 nm)/TCTA (5 nm)/46a or 46b/TmPyPB (55 nm)/LiF (1 nm)/Al, where, dipyrazino[2,3-*f*:2′,3′-*h*]quinoxaline-2,3,6,7,10,11-hexacarbonitrile (HATCN),1,1′-bis(di-4-tolylaminophenyl)cyclohexane (TAPC), 1,3,5-tri(mpyrid-3-yl-phenyl)benzene (TmPyPB), and TCTA were used as HIL, HTL, ETL, and exciton-blocking layer, respectively. The compound 46a showed color coordinates of (0.28, 0.58), along with *η*_ext_ value of 6.5%_._ Likewise, the compound 46b showed coordinates of (0.39, 0.57), along with a maximum *η*_ext_ value of 12.7% corresponding to a bright yellow emission. The delayed fluorescence (*Φ*_delayed_) and prompt fluorescence (*Φ*_prompt_) of 46a films were found to be 52.1 and 9.4%, respectively, which depend on the lifetime of the molecules. Also, the AIDF properties of the compound reduce the exciton annihilation and emission quenching in neat films.

#### 4.1.2 Star shaped hexarylbenzenes

**Fig. 10:**
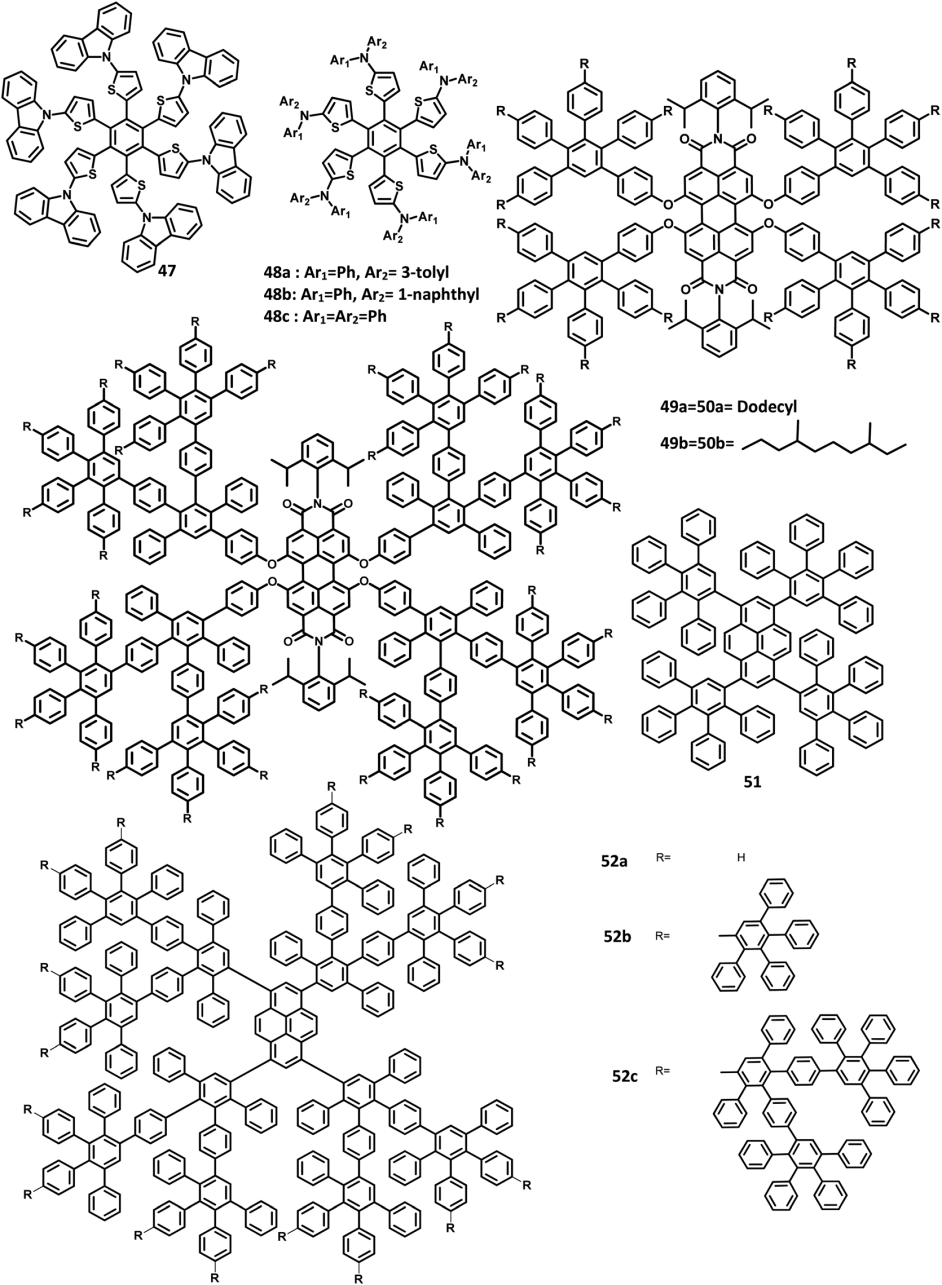
Molecular structure of HAB derivatives 47–52.

There have been extensive studies on “star-burst” dendrimers to achieve high device efficiency and durability. The dendrimers for EL applications are designed with conjugated and non-conjugated scaffolds for better charge transport and surface-to-core energy transfers, respectively.^106,119–123^ Lin and co-workers^124^ reported a thiophene-based hexaaryl derivative with conjugated scaffolds for OLED applications (Fig. 10). They have synthesized hexakis-[(diarylamino)thienyl]benzene using the palladium-catalyzed coupling reaction of hexabromobenzene. The six thiophene rings were introduced in the central benzene to reduce the oxidation potential of arylamines. The compound 47 is crystalline and has the highest *T*_d_ value among the reported polyhydrocarbons. The compounds 48a–c exhibited quasi-reversible peaks along with six-electron redox processes resulting from six diarylamine moieties. Moreover, the oxidation potential decreases in the order 47 ≫ 48b >48c > 48a, which may be justified by the electron-withdrawing effect of arenes to the nitrogen atom. To examine the EL properties of 47 and 48a–c, double layer device was fabricated with synthesized compounds as the HTL and Alq_3_ acting as the ETL as well as the emissive layer. All the devices showed green emission in the wavelength of 520 nm. The device exhibited turn-on voltage, maximum luminescence, and EQE values in the range of 6–8 V, 14 000–20 000 cd m^−2^, and 1.2–1.6%, respectively, which are comparable to the performance of the standard materials (7 V, 25 000 cd m^−2^, 1.3%).

The monochromatic OLED-based materials play a major role in the commercial industries as well as research on multi-chromatic materials. Mullen and team^125^ reported perylene diimide (PDI) chromophore-tethered HAB dendrimers for OLED applications (Fig. 10). All these dendrimers were prepared through palladium-catalyzed Sonogashira reaction followed by Diels–Alder cyclo-addition. This skeleton reduces the unwanted aggregation in the solid-state by shielding the chromophores. Subsequently, it was found that the dendrimers containing alkyl chains at the bay position exhibited excellent shielding compared to the imide segments. The absorption spectra of 49a, b and 50a, b were exhibited in two predominant regions, which are attributed to the central chromophore (585, 543, and 465 nm) and dendritic framework (∼278 nm). The absorption at the dendron region and solubility of the dendrimers increased with the increase in the number of dendrons consisting of twisted benzene rings. To evaluate the effect of dendron, the EL device was fabricated with the following configuration of ITO/(1.3wt%) PEDOT:PSS (70 nm)/50a or 49b (100 nm)/Al (120 nm). The lowest *T*_m_ value was observed for 49a and thus, the molecule is not used for fabricating the EL device. Surprisingly, the molecules with more dendrons (50b) were observed with a blue-shifted EL spectrum and a turn-on voltage of 9 V, which is higher than that of 49b (4 V). The poor performance of 49b is attributed to the weak charge transport behavior of the 2G dendritic segments. Besides, there was a simultaneous reduction in the EL intensity and current density of the molecule, along with poor orange-red emission. This suggested that the charge transport properties are reduced concerning the increasing number of dendron segments within the core. To investigate the energy and charge transfer properties, the device was fabricated with PVK:PBD-based polymer matrix as the host layer. The EML containing 49a, b and 50a, b blended with this charge transport polymer matrix led to exciton formation by two pathways: (i) formation of exciton on the polymer matrix followed by the transfer of the resultant energy to the dendron core or (ii) trapping of the charge carrier on the emissive material. It was found that the exciton generation decreased the Forster transfer rate by a value of 1.3–1.8 (49a, 50a-1G and 49b, 50b-2G), which was consistent with the predictions of the “Forster transfer theory”.

Further studies by Müllen and co-workers^126^ proposed a set of pyrene-based dendrimers for OLED applications. These molecules containing a pyrene core was encapsulated with an HPB polyphenylene dendron, which acts as chromophore as well as electrophore. Four generations of dendrimers were prepared from a series of [4 + 2] cycloaddition reactions and 51, 52a, 52b, and 52c are the first (1G), second (2G), third (3G), and fourth (4G) generation dendrimers, respectively (Fig. 10). The absorption spectra of the compounds were observed in two main spectral ranges: (i) the band at high-energy UV region (280–350 nm) is attributed to the polyphenylene dendrons, whose intensity increased with the increasing generation, and (ii) the band at low-energy visible region (∼395 nm) are predominantly resulting from the π–π* transition of the pyrene core. The emission spectra were observed at ∼420 nm, showing no shift in the wavelength upon the variation of the polyphenylene dendron. The excitation of the pyrene core and polyphenylene dendron resulted in the appearance of bands within the same region, which denotes the efficient energy transfer between them. Furthermore, these four-generation dendrimers showed *Φ* > 90%, with 52a showing a good film-forming property in the solid-state. Thus, these new dendrimers may find applications in OLEDs.

**Fig. 11:**
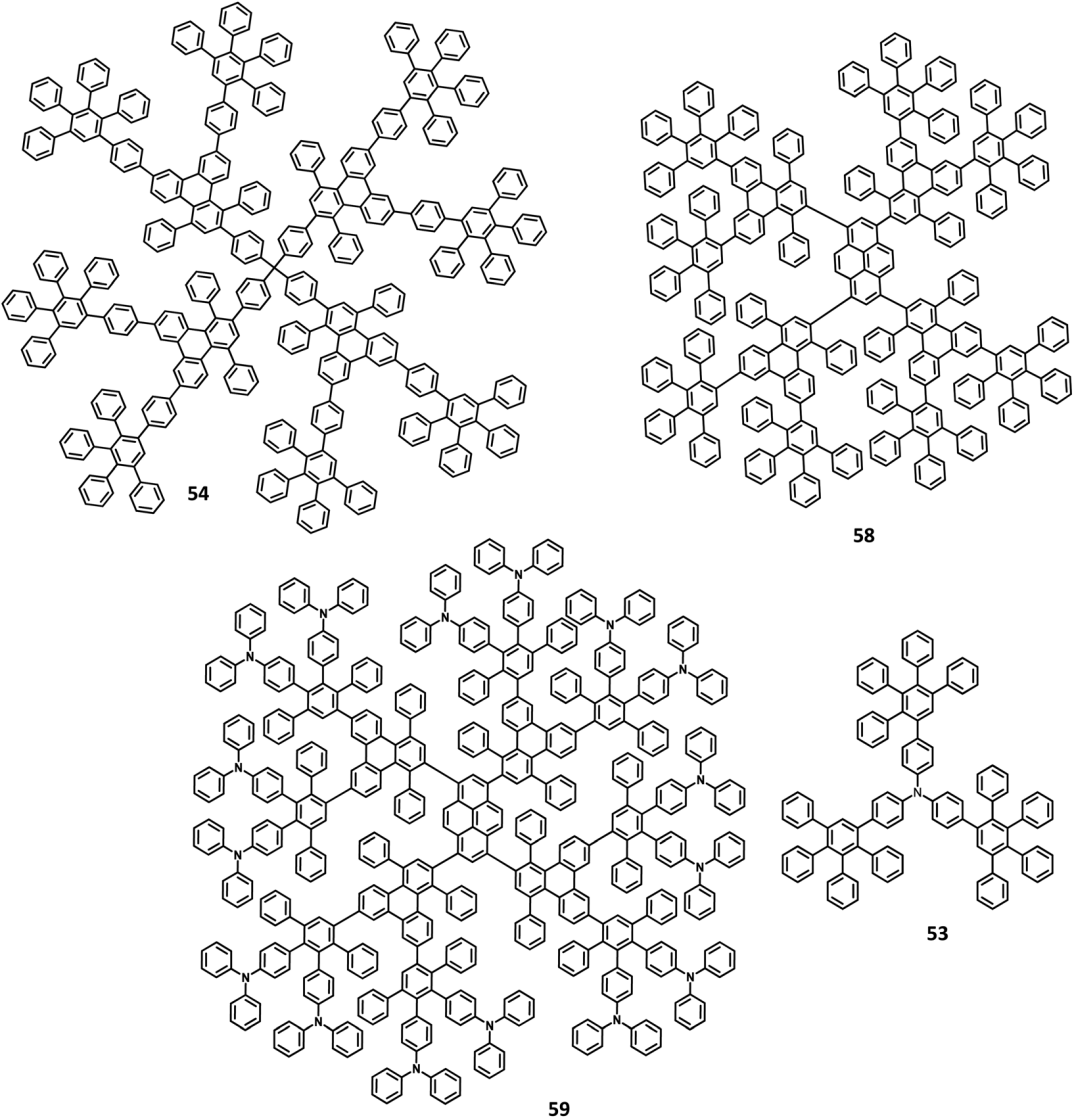
Molecular structures of HAB derivatives 53, 54, 58, 59.

Kwong and co-workers^127^ reported triarylamine-based PAB molecule 53 as a hole transporting material for green OLEDs (Fig. 11). Bulky substituents were introduced to design molecular glasses with high *T*_g_ values as the rotational, translational, and vibrational motions of the molecules are hindered. Tetraphenylphenylene moiety is an effective building block for the reduction of π–π aggregation in the solid-state. The optical bandgap of 53 was observed to be 3.1 eV and the HOMO and LUMO values were determined to be 5.4 and 2.3 eV, respectively. These properties indicate that 53 has comparable properties with the conventional NPB molecule. The OLED device fabricated with the synthesized 53 molecules and NPB in the following configuration as (i) ITO/NPB/Alq_3_/LiF/Al and (ii) ITO/53/Alq_3_/LiF/Al. Both the devices emitted green light, which indicates the hole–electron recombination occurring in the Alq_3_ molecule. Besides, there is no exciplex formation observed at the interface. The maximum brightness and turn-on voltage of the 53-based device were determined to be 2.54 V and 22 660 cd m^−2^, respectively. These are almost similar to the NPB-based device (maximum luminescence = 15 320 cd m^−2^ and turn-on voltage = 2.54 V). The device containing 53 showed maximum power and current efficiencies of 4.26 lm W^−1^ and 5.3 cd A^−1^, respectively, which are significantly higher than those of the standard device (3.0 cd A^−1^ and 2.92 lm W^−1^). This significantly improved the brightness and efficiency of the 53-based device due to the improved and balanced charge recombination at the EML interface. Furthermore, the compound 53 exhibited the hole mobility of 5 × 10^−5^ cm^2^ V^−1^ s^−1^, which is almost similar to Alq_3_ (10^−5^ cm^2^ V^−1^ s^−1^) and lesser than the NPB (10^−3^ cm^2^ V^−1^ s^−1^). The decreased current density of 53 is attributed to the decreased hole current as well as moderate mobility.

Another interesting multi-chromophoric dendrimer was synthesized due to its potential for application in the field of dendrimer light-emitting diodes (DLED). Mullen and co-workers^128^ reported giant-like multichromophoric dendrons tailored from PAB-attached polyhydrocarbon units that were used as blue emitters. Here, the segments were tailored into a rigid polyphenylene dendrimer and thus were endowed with a sterically free scaffold to inhibit the intermolecular chromophore interactions. Consequently, it results in a better amorphous propensity due to this interaction. The dendrimer 54 was synthesized *via* Diels–Alder reaction and showed absorption maxima of 299 nm in solution and 313 nm in the solid-state (Fig. 11). There is no change in the PL spectrum before and after the annealing process at 200 °C. The electroluminescent device composed of 54 was fabricated according to the following configuration: ITO/PEDOT:PSS/54/TPBI/CsF/Al, where TPBI serves as an additional electron transport layer to prohibit the charge recombination at the cathode interface. The electroluminescence spectrum resembles the PL spectrum of the molecule with the emission of deep blue color. This dendrimer showed color coordinates of (0.17, 0.10) along with a maximum luminance of 400 cd m^−2^ at a driving voltage of 10 V. The performance of this blue fluorescence device is comparable to the best reported dendrimer-based OLED device.

**Fig. 12:**
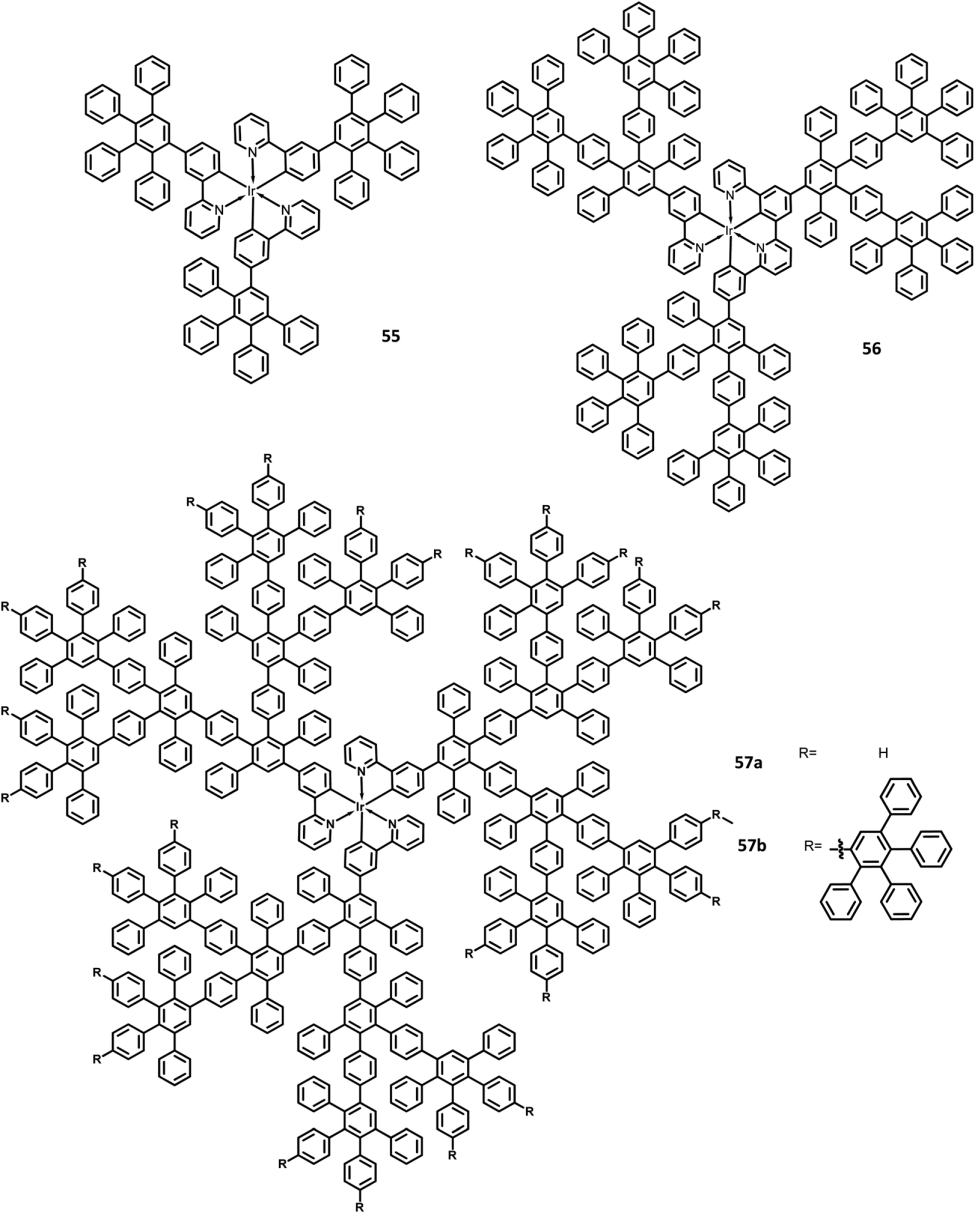
Molecular structures of HAB derivatives 55–57.

The development of an OLED device requires the prevention of aggregation in the solid-state. This is achieved by employing a matrix-chromophore blend rather than the chromophore alone. Although there are some limitations in the determination of distribution and arrangement of the chromophore and polymer, the intermolecular interactions within the dendritic architectures can suppress this behavior. Müllen *et al.*^129^ reported giant higher generation polyphenylene dendrimers forming complexes with Ir to investigate the effect of molecular size on the performance of phosphorescent OLED device. A series of four dendrimers 55, 56 and 57a, b with diameters of up to 8 nm and higher yields compared to the previously reported results were prepared (Fig. 12). The observed triplet–triplet annihilation from the Ir complexes was prohibited by the dendritic HPB segments present in the molecules, along with the enhancement of the quantum yield of the compounds. Besides, these dendrimers showed phosphorescence at room temperature and a drastic increase in the quantum yield of the complexes compared to the non-dendritic complex, with an enhanced solid-state phosphorescence for the 57b dendrimer. The color of the Ir(iii) complexes was tuned by appending the dendritic segments, which includes the incorporation of different homolepticcyclo metallated ligands and multicolor chromophores into the dendrimers. The absorption spectra of the lower dendritic complexes 55 and 56 were observed within the same regions, with the higher energy regions (300 nm) attributing to the π–π* transition and lower energy region ∼350–450 resulting from the metal to ligand charge transfer (MLCT). However, the MLCT is not observed in higher dendritic complexes due to the high absorption intensity of the dendritic segments. The emission spectra of these complexes were observed in the green emission region along with an aggregation-induced redshift for the lower dendritic complexes. The HOMO and LUMO levels of the complexes (55 and 56) were determined to be around ∼−5.0 eV and ∼−2.6 eV, respectively, with a bandgap of ∼2.4 eV. To investigate the structure–size relationship and electroluminescence properties, the following non-doped PhOLED device was fabricated with the architecture of ITO/PEDOT:PSS/55 or 56 or 57a or 57b/TPBI/LiF/Al. The dendrimer 57a exhibited a lower turn-on voltage than other dendrimers. It also showed maximum luminescence, luminescence efficiency, and the highest EQE values of 5900 cd m^−2^, 21.9 cd A^−1^, and 6.1%, respectively. To improve the performance of the device, different weight percentages of the dendrimer was used to fabricate the EL device. The EQE and maximum luminescence of the 56 (10.3%, 37.0 cd A^−1^) and 54 (10.2%, 36.5 cd m^−2^) were found to be better than those of 55. However, the doped and non-doped device performance of 57b was relatively low due to the existing twisted and extended polyphenylene segments that interrupt the charge transport distribution. Therefore, despite the high quantum efficiency of 57b, the device showed poor performance.

Mullen and team^120^ designed and synthesized polyphenylene-appended perylene dendrimers with a multifunctional core–shell surface structure for application in blue light-emitting diodes. Here, pyrene was taken as the core chromophore while triphenylamine was incorporated as the surface chromophore (Fig. 11). Besides, this structure was implemented to increase the photoluminescence quantum yield and obtain a wide range of emissions. Also, the polyphenylene groups increase the solubility of the dendrimer and thus enhance the thermal stability, providing a good amorphous propensity for the films. These dendrimers were prepared from a series of reactions, primarily involving [4 + 2] cycloaddition and TIPS desilylation reactions to furnish good yields. To locate the absolute energy levels, the HOMO levels of the pyrene core (5.7 eV) and triphenylene segments (−6.0 eV) were calculated. The triphenylamine segments facilitate hole injection from the PEDOT:PSS to the dendrimer 59. Hence, the device fabricated with 58, 59 as the HTL in the configuration of ITO/PEDOT:PSS/58 or 59 (30 nm)/TPBI(10 nm)/CsF (8 nm)/Al, where the heterostructured PEDOT:PSS/58/TPBI was taken as the electron transport layer for the device. Thus, 59 exhibited good stability, the highest current efficiency of 0.26 cd A^−1^, the highest luminescence of 1140 cd m^−2^, and CIE coordinates of (0.15, 0.17). The excellent performance, stability, and deep blue emission can be ascribed to the design of HAB derivatives, which furnish different properties in the solid-state.

**Fig. 13:**
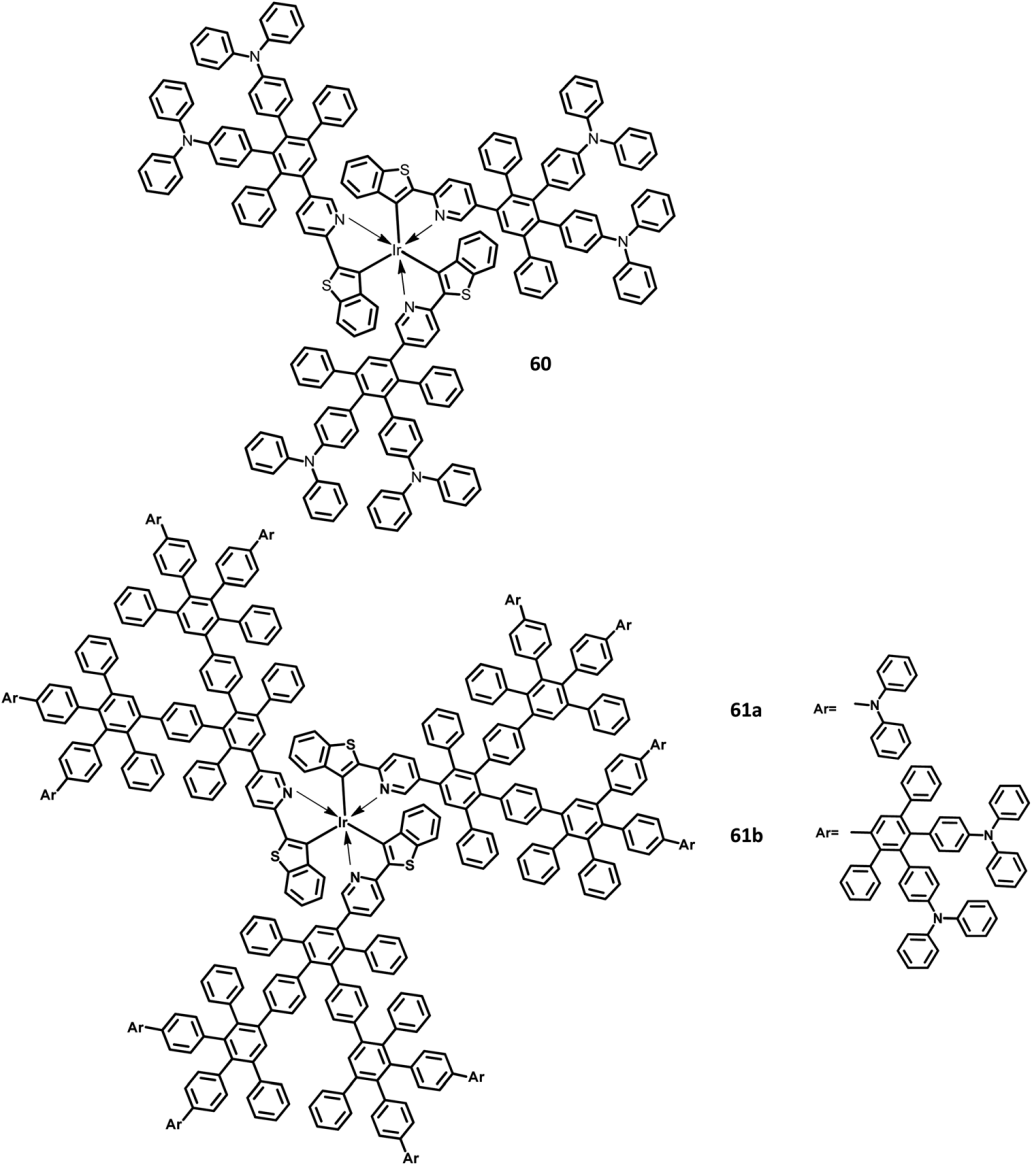
Molecular structures of HAB derivatives 60, 61.

Furthermore, Müllen and co-workers^119^ modified the HPB-appended Ir(iii) complexes by substituting the triphenylamine segments for red phosphorescent EL applications. These hole-transporting triphenylamine segments improve the PL and EL efficiencies by enhancing the charge recombination process and prohibiting the intermolecular AIE quenching in the solid-state. The first (60), second (61a), and third (61b) generation dendrons are prepared by stepwise synthetic procedures of Diels–Alder cycloaddition followed by Pd-catalyzed coupling reactions (Fig. 13). All the three dendrimers exhibited similar absorption spectra primarily in two regions: (i) the predominant band observed at higher energy regions (300–350 nm) ascribed to “spin allowed ligand-centered transitions” from the dendrons and (ii) the low-intensity band (483 nm for 61b and 423–424 nm for 60, 61a) observed at lower energy regions attributed to the MLCT band of the Ir(iii) complex to the dendrons. The inner chromophoric core with outer twisted triphenylamine segments facilitates the hole transport and injection ability. Similar to previous work, these dendrimers form good films. Consequently, the EL performance of these films was evaluated by fabricating the device with the configuration of ITO/PEDOT:PSS (50 nm)/(TCCz):60 or 61a or 61b (*x*%) (50 nm)/BCP (20 nm)/Alq (30 nm)/LiF (1 nm)/Al (100 nm), where Alq (tris(8-hydroxyquino) aluminum) is used as the HBL and TCCz (*N*-(4-[9,3′;6′,9′′]tercarbazolyl)phenylcarbazole) is used as the hole-transporting host material. The higher current density was observed for the dendrimers 60 and 61a rather than the 3D dendrimer under the same voltage, which is attributed to the enhancement of charge carrier mobility with increasing dendron segments/generations. The EL spectrum observed for the triplet excited state of the complexes indicated the emission of red light with the color coordinates of (0.68, 0.32) for 60, (0.66, 0.32) for 61a, and (0.63, 0.32) for 61b.

**Fig. 14:**
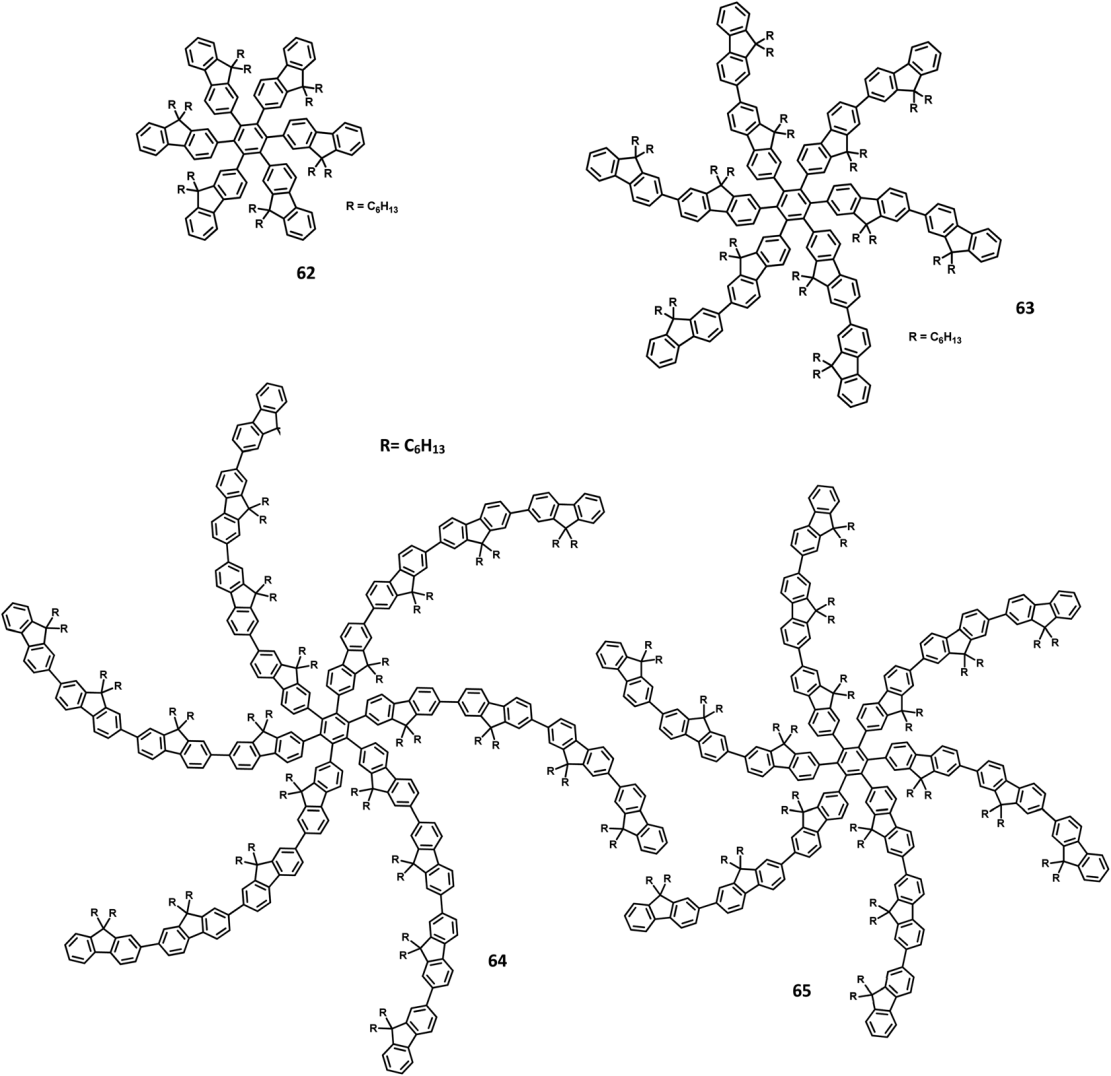
Molecular structures of HAB derivatives 62–65.

Yang and team^130^ reported that the HAB-tailored fluorene dendrimers can be utilized as high-efficiency emitters and hole-transporting materials for deep-blue electroluminescent devices. These molecules consist of an extremely twisted hexakis(fluoren-2-yl)benzene core attached with six oligofluorene forms 1D 63 to 3D 65 (Fig. 14). The absorption spectra of the dendrimers 63–65 in the solution showed sharp bands of π–π* transition (313–365 nm). The compounds 63–65 exhibited deep blue emission in both solid and solution states along with excellent *Φ*_PL_ values (0.88–0.92) in the solution. The OLED device was fabricated with dendrons using the following configuration: (ITO)/(PEDOT:PSS) (50 nm)/63 or 64 or 65 (70 nm)/(TPBI) (30 nm)/Ba (4 nm)/Al (150 nm), where TPBI is used as both HTL and HBL, and PEDOT:PSS is taken as the HIL. All the above devices were observed to have deep-blue emission behavior and CIE coordinates of (0.17, 0.08) for 63, (0.16, 0.08) for 64, and (0.16, 0.07) for 65, which are very close to the NTSC standards. The devices fabricated with 64 and 65 showed similar EL and PL spectra and good color stability as the voltage was increased from 4 V to 11 V. This is attributed to the suppression of close-packing, inhibition of morphological transition-induced deterioration, and resistance in crystallization observed in the solid-state that are induced by the increasing number of fluorene segments in the dendrimers. Furthermore, a high turn-on voltage was observed for these devices, owing to the high hole injection barrier between the HTL and EML. Besides, the dendrimers 63 and 64 exhibited better EL performances compared to 62 along with better color stability compared to the previously reported fluorine-based OLED devices. These dendrons exhibited *η*_ext_ and maximum luminescence values of 6.1% and 998 cd m^−2^ for 63, 6.7% and 1707 cd m^−2^ for 64, and 6.8% and 1962 cd m^−2^ for 65, respectively. Additionally, the hole transporting behavior of the dendrimers were evaluated by fabricating the EL device as follows: ITO/PEDOT:PSS (50 nm)/62 or 63 or 64 or 65 (45 nm)/Alq_3_ (60 nm)/LiF (1 nm)/Al (100 nm), where Alq_3_ served as the EML and dendrimers acted as the HTL. All the devices containing 62–65 exhibited emission spectra with maxima at 545 nm from the value of Alq_3_. The maximum current efficiencies of the above devices were determined to be in the range of 5.51–6.62 cd A^−1^, which are comparable with the standard device designed with NPB as the HTL (4.07 cd A^−1^). Furthermore, these star-shaped dendrimers showed lower hole mobilities compared to NPB (HTL). Consequently, the hole and electron fluxes were observed to be more balanced.

**Fig. 15:**
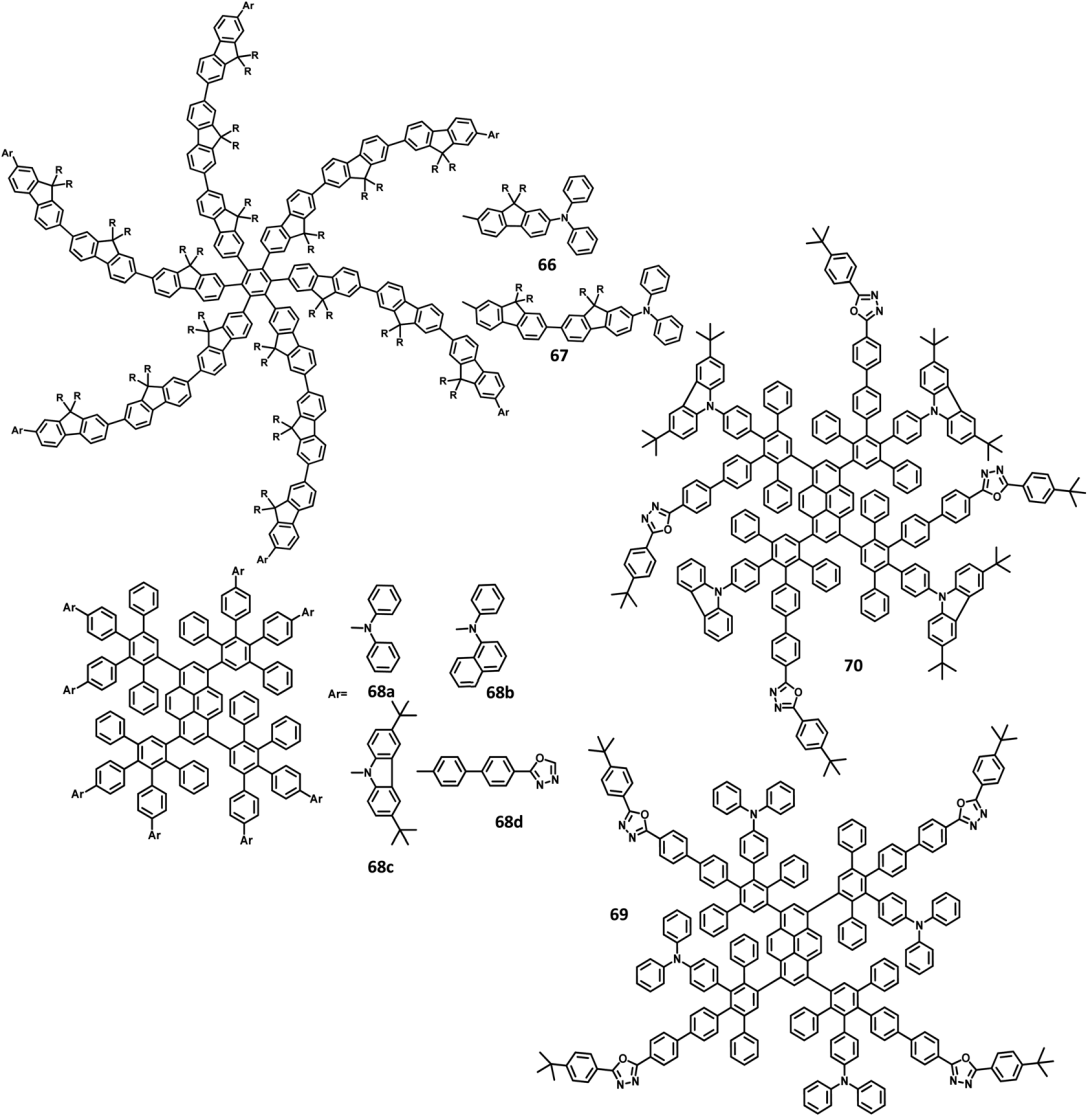
Molecular structure of HAB derivative 67–70.

A wide bandgap and high HOMO levels are prerequisites for efficient deep blue-emitting OLEDs. However, the previous reports (62–65) were found to achieve low HOMO levels, which led to low device performance such as low luminescence, device efficiency, and high *V*_on_. To address this issue, the same research group introduced triphenylamine into the dendrimer segments as an end-cap to raise the HOMO level. Yang and team^106^ were able to design and synthesize HPB-appended oligofluorene segments end-capped with triphenylamine unit for OLED applications (Fig. 15). To examine and compare the EL properties of these dendrimers, the OLED devices were fabricated with the configuration of (i) ITO/PEDOT:PSS_4083_ (40 nm)/66 or 67, (50 nm)/TPBI, (30 nm)/LiF, (1 nm)/Al, (100 nm). These emitters showed deep blue emission with the CIE color coordinates of (0.149, 0.104) and (0.151, 0.101) for 66 and 67, respectively. Besides, they showed independent EL behaviors towards voltage. Furthermore, these devices showed maximum luminesce and EQE values of 9524 cd m^−2^ and 5.30% for 66 and 8596 cd m^−2^ and 3.70% for 67, respectively. These values are higher than those of the dendrimers without end-capper, which is attributed to the increased hole injection from PEDOT:PSS to the emissive layer. To further increase the hole injection efficiency, the OLED device was fabricated with different PEDOT:PSS layers such as (ii) ITO/PEDOT:PSS_4083_ (40 nm)/66 or 67, (50 nm)/TPBI, (30 nm)/LiF (1 nm)/Al (100 nm). The EQE and maximum current efficiency values were observed to be 5.45% and 6.99 cd A^−1^ for 66 and 4.86% and 6.73 cd A^−1^ for 67, respectively. Thus, the rationalized device configuration and high HOMO level influence the device performance.

Müllen *et al.*^131^ reported polyphenylene-appended pyrene core molecules for application in deep blue OLEDs through surface-to-core energy transfer. These pyrene-based polyphenylene dendrimers were synthesized through cycloaddition reactions in good yields. The remarkable bathochromic shift was observed in the emission spectrum for all the compounds except 68b, which may be attributed to the presence of carbazole unit as the end group/surface group that shields the core (Fig. 15). The photoluminescence *Φ* values of the core and surface were observed to be ∼70% and ∼60%, respectively. The HOMO levels of the surface chromophore unit and core were found to be in the same energy levels for 68a (similar to triphenylamine) and 68b (similar to carbazole). By relating the work function of the buffer layer PEDOT:PSS and emissive layers, it is found that the charge injection ability of the dendrimers follows the order 68c > 68a > 68b ≫ 68d. These compounds exhibit an efficient “surface to core energy transfer”, which results in high-efficiency blue emission that possesses high *Φ* values. The device was fabricated in the following architecture: ITO/PEDOT:PSS/68a or 68b or 68c or 68d/Ca/Al. The dendrimer 68b showed comparatively better performance with the maximum luminescence value of 715 cd m^−2^ and high current efficiency of 0.24 cd A^−1^, which are attributed to better charge injection within the system. Due to the high energy injection barrier of Ca and chromophores, the following three-layer device configuration was used to fabricate the device as ITO/PEDOT:PSS/TFB/68a or 68b or 68c or 68d/PEGPF/Cs_2_CO_3_/Al, where PEGPF ((9,9-bis(3-(5′,6′-bis(4-(polyethyleneglycol)phenyl)-[1,1′:4′,1′′-terphenyl]-2′-yl)propyl)-9′,9′ dioctyl-2,7-polyfluorene) was used as the HTL and TFB (poly(9,9-dioctyl-fluorene-*co-N*-(4-butylphenyl)-diphenylamine)) was taken as the ETL. The dendrimers showed significantly higher performance than a single-layered device, with the 68a dendrimer particularly exhibiting an enhancement in the current efficiency by 70 times due to the decreased electron injection barrier. Also, 68b exhibited the highest current efficiency of 0.52 cd A^−1^, while 68c displayed the maximum luminescence of 3726 cd m^−2^.

Recently, Müllen and team^132^ reported two dendrimers with the electron as well as hole-transporting properties for blue OLED applications. This bipolar behavior results from the HPB-appended carbazole and oxazole segments used as dendrons that are connected to the pyrene core (Fig. 15). Thus, the dendrimer 69 exhibited deep blue emission with dual transport from the core chromophore resulting from the ICT behavior. Alternatively, the dendrimer 70 containing a carbazole surface group manifested a pure blue emission without notable ICT behavior. The crystal structures revealed that the torsional angle between the central chromophore and polyphenylene segments at the 3,4 positions were observed to be 53° and 48° for 70 due to the steric hindrance between the units. Compared to the bare units, slightly higher values were observed for the BPD segments due to the reduced conjugation of BPD with the central chromophore. The emission band was observed at 431 nm with an additional shoulder peak that originates from the excitation values (core and surface of the carbazole or triphenylamine), which confirms the core-to-surface energy transfer. As per our knowledge, these are the first dendrimers containing pyrene core that were reported to exhibit bipolar transport properties. The HOMO–LUMO energy levels can be tuned using shape-persistent dendrimers. The LUMO of the dendrimers 68a and 68b were observed at around ∼2.4 eV, which are similar to other reported pyrene-based dendrimers. The HOMO of the dendrimers 68a and 68b were determined to be −5.34 and −5.52 eV, respectively. These values are close to the energy level values of the corresponding triphenylamine and carbazole moieties, which indicate the slight conjugation of the core to the surface group due to the low torsional angle. To evaluate the electroluminescence performance, the derivative 68b was incorporated in single-, double-, and triple-layer EL devices while the other derivatives were employed in either single- or double-layer devices. The device follows the configuration of (i) ITO/PEDOT:PSS/68a or 68b or 70/Ca/Al; (ii) ITO/PEDOT:PSS/TFB/68b/Ca/Al; and (iii) ITO/PEDOT:PSS/TFB/68b/PEGPF/Ca/Al. The three-layer device incorporated with dendrimer 68b showed better performance with the maximum luminescence of 2701 cd m^−2^ and efficiency of 0.21 cd A^−1^, which is ascribed to the improved electron injection of the multilayer device compared to the single- and double-layer devices. Due to the ICT behavior, the other dendrimer showed poor performance compared to 68b, and were not used for further investigation.

**Fig. 16:**
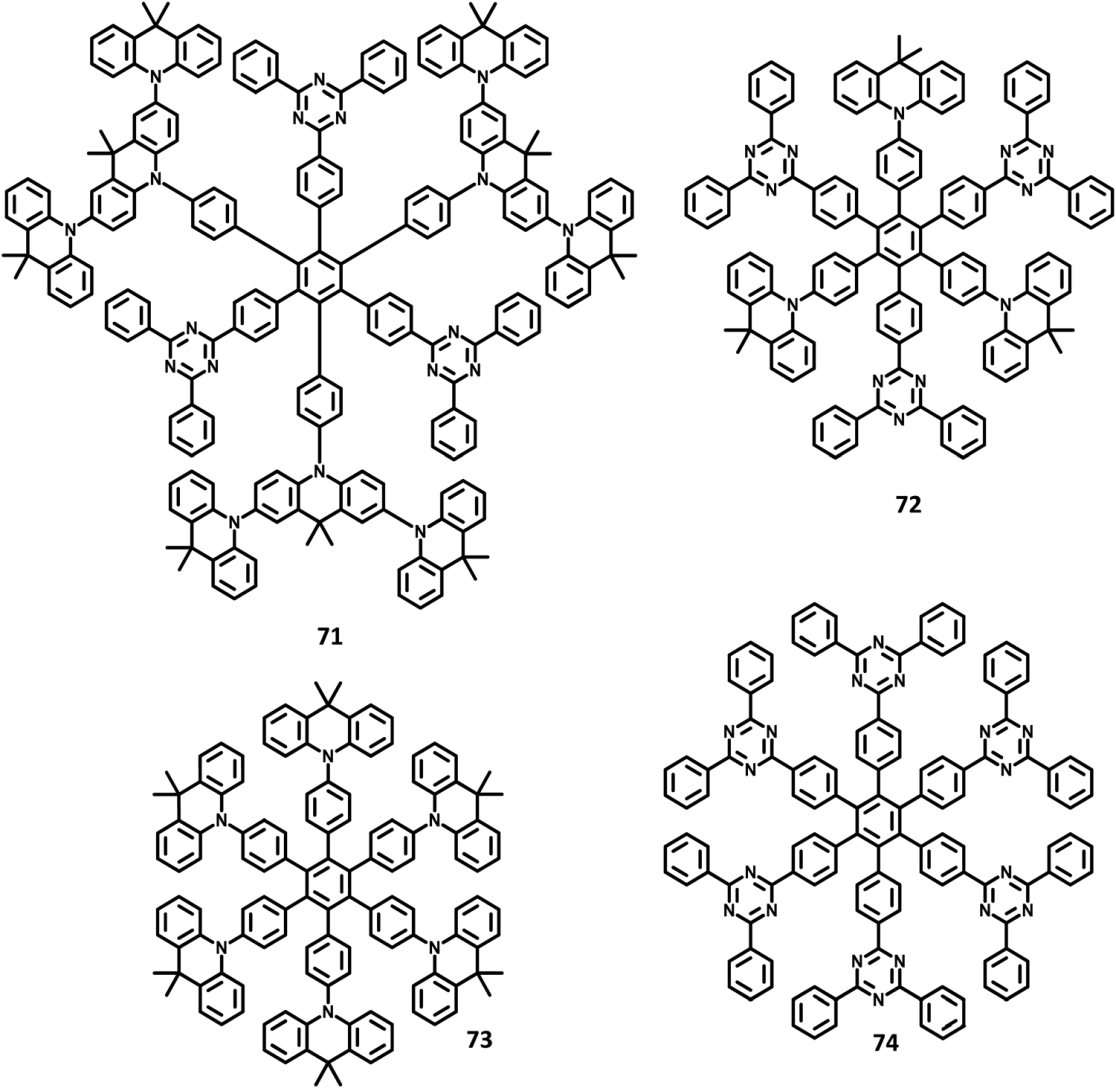
Molecular structure of HAB derivative 71–74.

Very recently, another interesting report with a donor–acceptor system utilizing AIE and TADF phenomena, Wang *et al.*^133^ studied solution-processable OLEDs and reported the influence of AIE and thermally activated delayed fluorescence phenomena on the regulation of emission behavior. In their work, circularly-arrayed dendrimers 71 and 72 containing electron donors (acridan) and acceptors (triazine) were designed, wherein each arm is closed around the central benzene to investigate ‘through space charge transfer’ (Fig. 16). Besides, two kinds of model dendrimers attached to only donors (73) and acceptors (74) were designed to explore the origin of CT emission. These molecules were synthesized through cyclotrimerization followed by Suzuki coupling reactions. Based on the promising photochemical properties of the molecules, a non-doped OLED device was fabricated with the following configuration: ITO/PEDOT:PSS (40 nm)/71 or 72 (40 nm)/TSPO1(8 nm)/TmPyPB (42 nm)/LiF (1 nm)/Al (100 nm), where, TmPyPB^133^ represents 1,3,5-tri(*m*-pyrid-3-yl-phenyl)benzene and TSPO1^134^ denotes diphenyl(4-(triphenylsilyl)phenyl)phosphine oxide. The non-doped devices (neat films of 71 or 72) show maximum EQEs of 3.1% and 3.5% for 71 and 72, respectively. Also, the maximum luminescence efficiencies of 10.2 cd A^−1^ and 11.4 cd A^−1^, respectively, were observed. Interestingly, the doped devices were found to exhibit enhanced performance (EML with 73) with maximum luminescence of 40.6 cd A^−1^ and 30.3 cd A^−1^ for 71 and 73, respectively. Among them, the 71 molecule showed a maximum EQE of 14.2%, which is higher than that of the non-doped devices. These TSCT-HAB derivatives can be potential candidates for enhancing the efficiency of the emitters that exhibit AIE and TADF phenomena.

#### 4.1.3 Polymer analogues of hexaarylbenzene

**Fig. 17:**
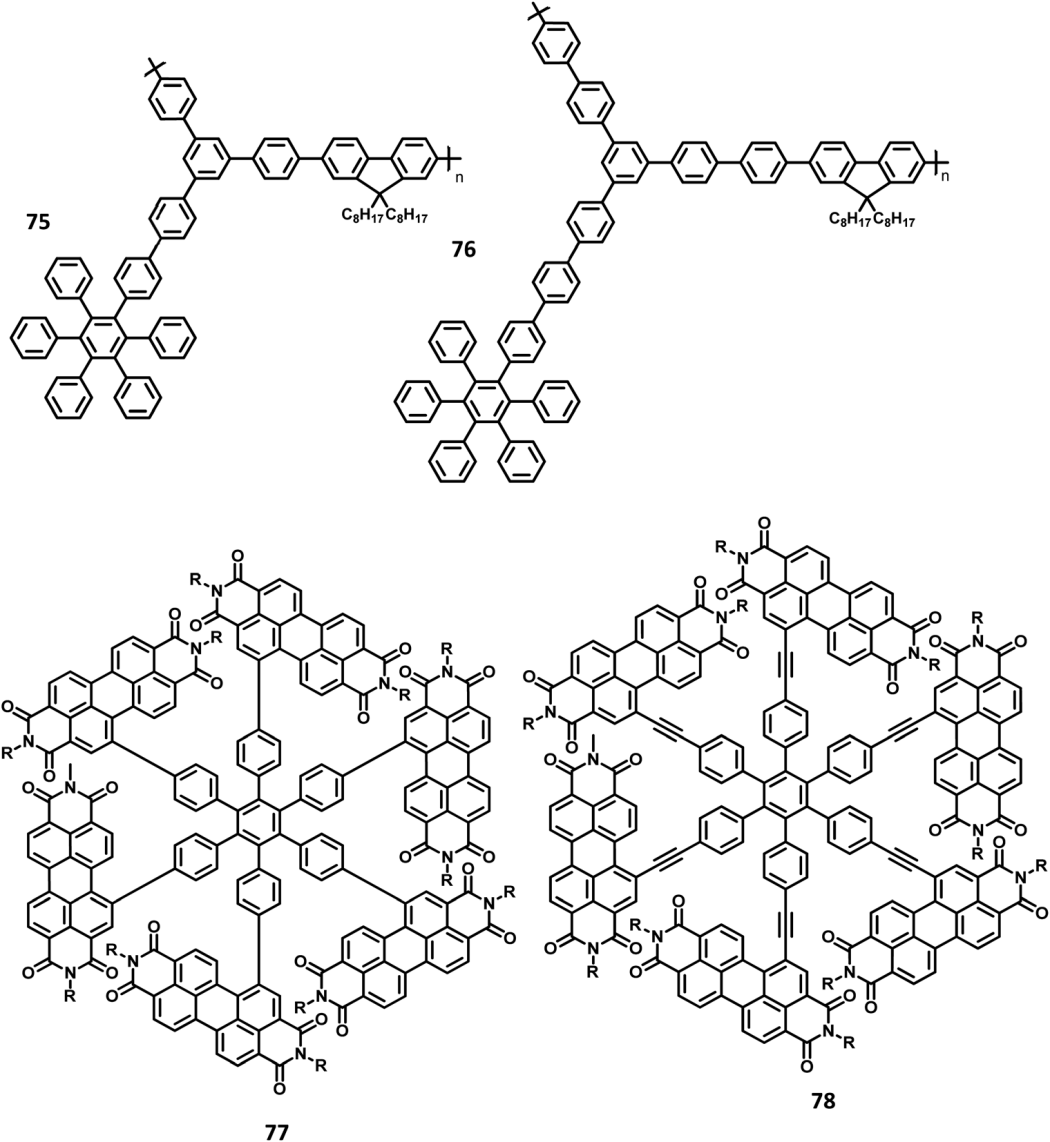
Molecular structure of HAB derivatives 75–78.

Liaw and co-workers^68^ reported the development of unsymmetrical HPB-appended poly(triphenylamine-*alt*-fluorene) polymers for NIR light-emitting diodes. The polymers 75 and 76 were synthesized *via* Suzuki polymerization of a monomer unit prepared using the Buchwald–Hartwig amination reaction (Fig. 17). The HOMO and LUMO energy levels of 75 and 76 were estimated to be −5.07 and −2.35 eV, respectively. The polymer film coated graphene-poly(ethylene terephthalate) (PET) or ITO plates exhibited color change on increasing the applied voltage. At 0 V, the polymer-coated ITO and PET films exhibited absorption at 1231 nm (NIR region), while additional peaks were observed at 510 and 1030 nm. Also, the color changes occurred from yellow to blue on increasing the potential. This electrochromism originates from the triphenylamine and HPB segments due to radical delocalization. Thus, HPB incorporation has an important role in enhancing the NIR absorption. The desirable properties suggest the applicability of these polymers in light-emitting optoelectronic devices.

### 4.2. Organic photovoltaics

#### 4.2.1 Organic solar cells

Recently, OSCs have been employed as a successful photovoltaic technology due to easy processability, realistic power conversion efficiencies, and cost-effectiveness.^135^ The tremendous progress in this field is attributed to the operation principles of the OSCs. The interfacial layers in the OSCs possess multiple functions in the devices.^136^ In general, a donor and acceptor with elevated charge carrier mobilities, and small energy offsets are required to develop proficient photovoltaics with low voltage compensations. An OSC involves an active layer, charge transporting layers, and electrodes. The active layer is composed of p-type and n-type blends and serves as light-harvesting material that also permits charge separation.^137,138^ The light is absorbed by an active layer composed of a D–A system. The formed excitons diffuse into the interface of the D/A and dissociate into electrons and holes. The dissociated electrons and holes move to the cathode and anode through the channel, respectively. Two leading architectures are employed in OSCs, namely bilayer heterojunction and bulk heterojunction.^139^ The BHJ OSCs consisting of organic semiconducting D–A systems have attained great success in harvesting solar light and converting it to electrical energy. Though fullerene derivatives are leading acceptors, they possess several limitations such as the limited tunability of energy levels and structure, high manufacturing cost, and narrow spectral absorption that restrict their practical application.^140^ To overcome these drawbacks, non-fullerene acceptors were introduced to replace the fullerene derivatives. Furthermore, the advantages of the tunable optical and electronic properties with stable structural morphology retain demand for non-fullerene molecules. Though many non-fullerene molecules are explored, the over-aggregation and phase separation of these molecules in the active layer do not facilitate the exciton diffusion and separation.

Constructing an active layer with star-shaped/asymmetric structure and 3D network is an effective strategy to reduce the strong aggregation that results in appropriate domain phases to enhance the photovoltaic performance. The application of non-fullerene HAB-PDI materials in organic photovoltaics was pioneered by Liu and co-workers.^140^ The novel bulk-heterojunction D–A system achieved from utilizing poly[4,8-bis(5-(2-ethylhexyl)thiophen-2-yl)benzo[1,2-*b*;4,5-*b*′]dithiophene-2,6-diyl-*alt*-(4-(2-ethylhexyl)-3-fluorothieno[3,4-*b*]thiophene)-2-carboxylate-2-6-diyl]^141^ (PTB7-Th) as the electron donor material and star-shaped HAB-appended PDI units as the electron acceptor for OSCs. Improved electron mobility and suppressed charge recombination were observed in the active layer on increasing the dimensionality of the PDI. This twisted propeller structure reduced undesirable aggregation in the 77 blend film (Fig. 17). Consequently, the electron charge mobility and power conversion efficiency were increased. The absorption spectrum of 77 was observed at 542 nm along with an additional hump at a lower energy region (510 nm). The HOMO and LUMO of the compound were observed to be −5.69 eV and −3.93 eV, respectively, with a bandgap of 2.10 eV. The suitable energy levels and absorption spectrum of PTB7-Th suggest that the molecule is a donor while 77 is an acceptor. The BHJ OSCs were fabricated by following the architecture of ITO/ZnO (30 nm)/(PTB7-Th/77 blend)/MoO_3_ (8.5 nm)/Ag (100 nm). The device exhibited a *J*_sc_ value of 13.18 mA cm^−2^, FF of 43.5%, and PCE of 5.12% with a *V*_oc_ of 0.89 V. The improved performance of PCE (5.77%) and FF (47.8%) were detected along with an additive layer of 0.1 vol% DIO. The device performance was also improved by increasing the annealing temperature of the active layer. To study the charge carrier properties of the D and A, the hole and electron only devices were fabricated with the configuration of ITO/PEDOT:PSS/PTB7-Th/Au and FTO/77/Al, respectively. These devices exhibited hole mobilities *μ*_h_ of 2.70 × 10^−5^ cm^2^ V^−1^ s^−1^ and electron mobility *μ*_e_ of 1.46 × 10^−4^ cm^2^ V^−1^ s^−1^. Moreover, there was an increase (*μ*_h_ = 8.31 × 10^−5^, *μ*_e_ = 2.42 × 10^−4^ cm^2^ V^−1^ s^−1^) and balance in the mobilities (*μ*_h_/*μ*_e_ = 2.91) upon the addition of 0.1% DIO additive layer. The enhanced *J*_sc_ observed for the optimized devices originates from the balanced and increased charge mobilities of the active layers.

**Table 2:** Solar cell properties of HAB derivatives

	*λ*_max_	*E*gopt	HOMO/LUMO (eV)	*μ* (cm^2^ V^−1^ s^−1^)	Active layer	*V*_oc_ (V)	*J*_sc_ (mA cm^−2^)	FF	PCE (%)	Ref.
77	511, 542	2.10	−5.69/−3.93	c2.70 × 10^−5^	PTB7-Th:77/MoO_3_	0.89	13.18	43.5	5.12	140
77	511, 542	2.10	−5.69/−3.93	c8.31 × 10^−5^	aPTB7-Th:77, 0.10% DIO/MoO_3_	0.91	13.22	47.8	5.77	140
77	511, 542	2.10	−5.69/−3.93	c1.03 × 10^−4^	bPTB7-Th:77, 0.10% DIO/MoO_3_	0.92	15.11	48.0	6.63	140
78	410, 600	1.99	−5.85/−3.86	9.01 × 10^−5^	Zno/PBDB-T:78/MoO_3_	0.86	9.71	42.5	3.52	28
78	410, 600	1.99	−5.85/−3.86	1.46 × 10^−5^	dZnO/PBDB-T:78/MoO_3_	0.91	10.86	57.9	5.71	28
79	312		−5.31/−2.10	4.98 × 10^−4^	TiO_2_/PCBA/CH_3_NH_3_PbI_*x*_Cl_3−*x*_/79	1.13	22.90	68.5	17.73	32
80	367	3.10	−5.25/−2.10	1.02 × 10^−4^	ETL/MAPbI_3_/80	0.92	21.86	69.9	12.94	148
81	386	2.78	−5.33/−2.52	5.48 × 10^−4^	ETL/MAPbI_3_/81	1.03	22.79	73.7	17.29	148
79	306, 314	3.19	−5.22/−2.03	7.38 × 10^−4^	eTiO_2_/C-PCBSD Perovskite/79	1.09	20.3	71.6	16.0	27
79	306, 314	3.19	−5.22/−2.03	7.38 × 10^−4^	fTiO_2_/C-PCBSD Perovskite/79	1.11	21.1	76.4	18.0	27
82	387, 376	3.05	−5.19/−2.14	5.54 × 10^−5^	eTiO_2_/C-PCBSD Perovskite/82	1.08	19.9	69.0	14.9	27
82	387, 376	3.05	−5.19/−2.14	5.54 × 10^−5^	fTiO_2_/C-PCBSD Perovskite/82	1.09	20.3	75.4	16.7	27
83	∼350	—	−5.05/−1.96	—	d ^,^ fCompact TiO_2_/mesoporous TiO_2_/Cs_0.5_(MA_0.15_FA_0.85_)_0.95_Pb(I_0.85_Br_0.15_)/83	1.04	23.28	72.5	17.48	52
83	∼350	—	−5.05/−1.96	—	d ^,^ eCompact TiO_2_/mesoporous TiO_2_/Cs_0.5_(MA_0.15_FA_0.85_)_0.95_Pb(I_0.85_Br_0.15_)/83	0.988	23.14	57.5	13.1	52

a With 0.10% DIO additive.

b With 5 min annealing *T*.

c Calculated by ssdd method.

d With additive.

e Measured in forward scan.

f Measured in reverse scan.

Recently, another flower-shaped non-fullerene based acceptor was synthesized by Zhang and coworkers^28^ from a PDI-HAB core tailored ethynyl bridge. This flower-shaped framework can suppress strong aggregation and also develop a 3D-network similar to fullerene derivatives. This molecule was prepared *via* Sonogashira coupling reaction. The HOMO and LUMO of 78 were observed to be −5.85 and −3.86 eV, respectively (Fig. 17). The polymer poly[(2,6-(4,8-bis(5-(2-ethylhexyl)-thiophen-2-yl)-benzo[1,2-*b*:4,5-*b*′]dithiophene))-*alt*-(5,5-(1′,3′-di-2-thienyl-5′,7′-bis(2-ethylhexyl)benzo[1′,2′-*c*:4′,5′-*c*′]dithiophene-4,8-dione))] (PBDB-T) was chosen as the electron donor to fabricate the solar cell due to its suitable HOMO–LUMO energy levels and complementary absorption to 78. To evaluate the electronic properties of the 78 acceptor, the photovoltaic device was fabricated with the following architecture of ITO/ZnO/(PBDB-T:78) blend/MoO_3_/Ag. The optimized device exhibited a *V*_oc_ of 0.86 V, FF of 42.5%, and *J*_sc_ of 9.71 mA cm^−2^. Furthermore, the device was fabricated with an additive (1-chloronaphthalene, CN), which exhibited better device performance with a *V*_oc_ of 0.91 V, FF of 57.9%, and *J*_sc_ of 10.86 mA cm^−2^. Furthermore, the device containing CN additives showed good photo-response with an EQE of 76%, which is higher than the device without additive. The device containing CN showed excellent photoelectron conversion tendency. To study the charge transport properties, the pristine 78 and the blended active layer was fabricated using the device configuration of ITO/ZnO/78/Al and ITO/ZnO/(PBDB-T:100blend)/Al. The electron mobilities were found to be 1.46 × 10^−5^ and 9.01 × 10^−5^ cm^2^ V^−1^ s^−1^ for blend and pristine films, respectively. On examining the exciton dissociation energy, the blend film with CN showed 95% quenching efficiency compared to that without the additive. It indicates the higher excitation dissociation observed between the active blend films with CN, resulting in high *J*_sc_ for the device. Also, these films exhibit high thermal stability due to the twisted molecular geometry of 78, which may hinder over aggregation at high temperatures.

#### 4.2.2 Perovskite solar cells

Another famous class of photovoltaic technology is the perovskite solar cells (PSCs). The perovskite shows outstanding optoelectronic properties, such as wide absorption spectrum, high absorption coefficient, low exciton binding energy, high charge carrier mobility, as well as, long electron, and hole-diffusion length.^142^ Suitable hole/electron transport materials can regulate the bandgap of the perovskite material.^143–145^ Nowadays, mixed perovskites containing HTM exhibited the best results in device performance and stability. A gamut of materials was reported from the organic photovoltaic community, wherein the use of hole transport materials was intensely investigated.^142^ For effective application of these HTM in PSCs, its HOMO should be lower in energy than the perovskite VB. The HTMs play an important role by transferring the holes from the perovskite layer to the hole-collecting electrodes along with a reduction of hole–electron recombination by blocking the electrons. Star-shaped molecules showed multiple arms and 3D connectivity in the active layer, thereby acting as an efficient HTM for perovskite solar cells. Especially, the classic 2,2′,7,7′-tetrakis-(*N*,*N*-bis(4-methoxyphenyl)amino)-9,9′-spirobifluorene (spiro-OMeTAD) molecule has been widely investigated as an HTM containing four arms of dimethoxydiphenylamine.^146^ Apparently, the charge transport, hole mobility, thermal, photophysical, and electrochemical properties rely on the center core and branching segments. The HAB-based molecules were successfully utilized as HTM layers, owing to their good hole-transporting behavior and act as alternatives for the classic spiro-OMeTAD compound.

**Fig. 18:**
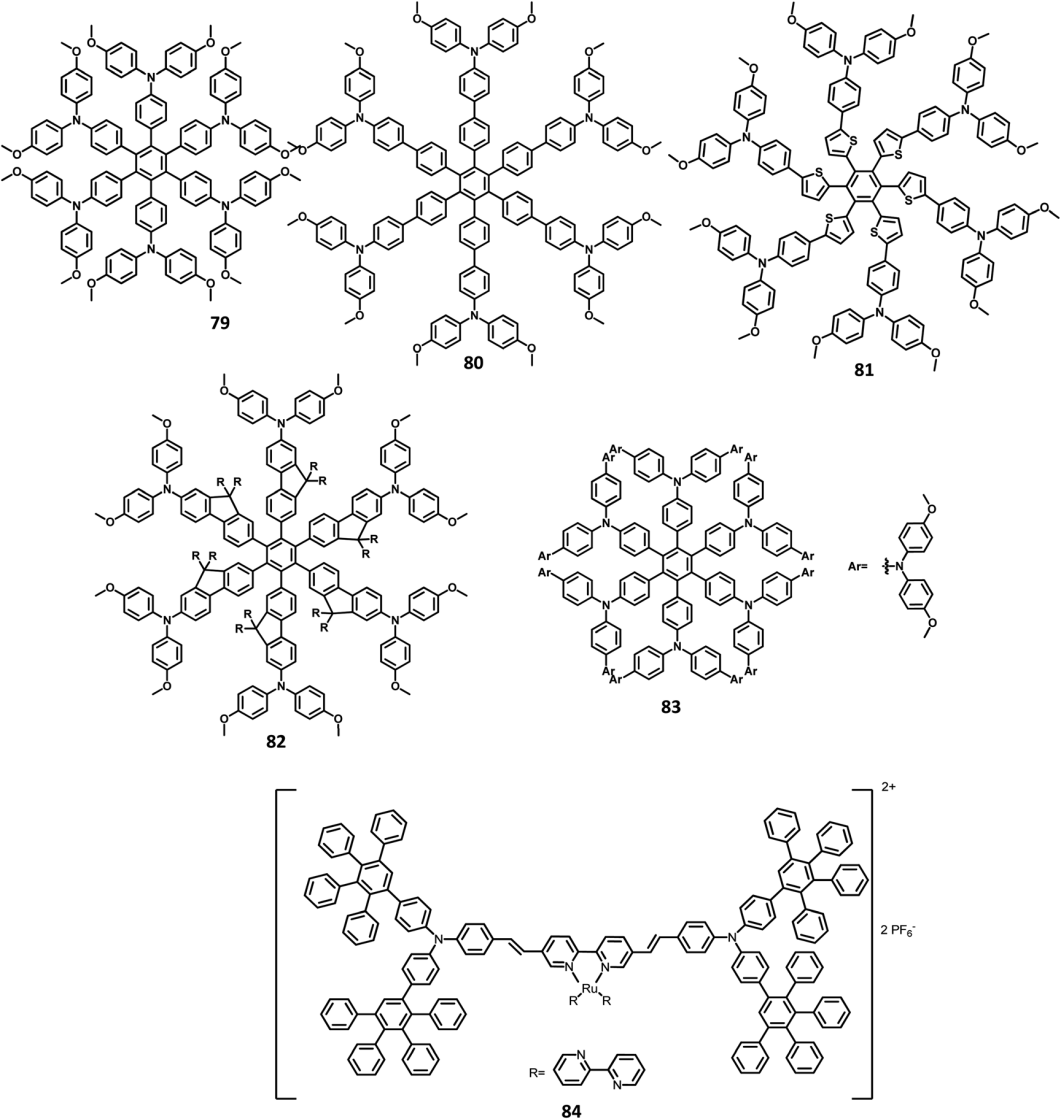
Molecular structure of HAB derivatives 79–84.

Li *et al.*^32^ synthesized a HAB appended triphenylamine based hole transport material *via* easy synthetic steps for PSCs. The derivative 79 was designed with six-hole transporting (bis(4-methoxyphenyl)-amino) segments (Fig. 18). Due to the suitable HOMO levels and high charge carrier mobility of 79 to the perovskite layer, it could be an excellent alternative for spiro-OMeTAD. The PSC was constructed in the configuration of FTO/TiO_2_ (130 nm)/PCBA/CH_3_NH_3_PbI_*x*_Cl_3−*x*_ (230 nm)/79/Ag, wherein, PCBA is [6,6]-phenyl-C_61_-butyric acid.^147^ To compare the results the same device configuration was fabricated with spiro-OMeTAD hole-transport material and the data are summarized in Table 2. Better energy agreement was observed between 79 and CH_3_NH_3_PbI_*x*_Cl_3−*x*_, which act as a driving force for the better charge injection. Therefore, the device showed a better *J*_sc_ of 22.90 mA cm^−2^ with a *V*_oc_ of 1.13 V and a fill factor of 68.5%. Besides, the device showed better performance than spiro-OMeTAD with an EQE of 17.73%. The molecule 79 was fabricated in hole only device to calculate the mobility as ITO/PEDOT:PSS/Au. The hole mobility of 4.98 × 10^−4^ cm^2^ V^−1^ s^−1^ was obtained for 79. Hence, such suitable electrical–optical properties were provided the appropriate energy level and superior charge transfer/extraction ability.

Cui *et al.*^148^ developed the non-fullerene triphenylamine tailored HAB derivatives for perovskite photovoltaic applications. Propeller-shaped hexaphenyl (80) and hexathienyl (81) benzene derivatives have been designed with triarylamine moiety, due to the good hole-transporting property (Fig. 18). HOMO energy levels were determined to be −5.25 eV for 80 and −5.33 eV for 81. Those energy levels matched with the perovskite layer which guarantees the effective hole injection and transmission between the light absorber and electrodes interface. The lower energy gap of the derivatives ensures that there can't be electrons backflow from the perovskite layer to HTLs (80 and 81). To study the electronic properties, the PSC was fabricated in the architecture of ITO/ETL (30 nm)/MAPbI_3_ (610 nm)/81 (60 nm)/Au (30–80 nm), where SnO_2_ serves as ETL. The dopant-free HTM layer thickness (∼20, ∼60, ∼100, and ∼200 nm) plays an important role in increasing device performance. The optimized device exhibited excellent PCE of 17.29%, *V*_oc_ of 1.03 V, *J*_sc_ of 22.79 mA cm^−2^and FF of 73.7%. On the other hand, the device with 80 exhibited a PCE of 12.94%, *V*_oc_ of 0.92 V, *J*_sc_ of 21.86 mA cm^−2^ and FF of 64.3%. The PL quenching studies reveal that the hole transfer from 80 to the perovskite layer is feasible, with hole mobility compared to 81. The hole mobilities of 80 and 81 were calculated to be 5.482 × 10^−4^ and 1.025 × 10^−4^ cm^2^ V^−1^ s^−1^, respectively. The better energy agreement of 81 with the valance band (VB) of the perovskite layer ensures better device performance.

Tao and co-workers^27^ reported a fluorene and triphenylamine appended HAB derivatives were utilized as HTM for PSC applications. The structure–electronic property relationship of HTMs was investigated by the research team. The 3D network structure along with better solubility was achieved from central benzene appended triphenylamine moiety. Due to different bridge groups, compound 79 and 82 were observed with different electrochemical, photophysical, thermal, and charge transport properties The HPB derivatives were exhibited HOMO and LUMO of −5.19 and −2.14 eV for 82 and −5.22 and −2.03 eV for 79, respectively (Fig. 18). These proper low-lying HOMO levels are suitable well with VB of CH_3_NH_3_PbI_3_ and work function of Au, thereby it favors the charge transfer at CH_3_NH_3_PbI_3_/79 or 82 interface. Furthermore, charge recombination was reduced by the prevention of electron transfer from the perovskite active layer to the anode. Besides, to measure the charge carrier properties, the hole-only device was fabricated in the architecture of ITO/PEDOT:PSS/79 or 82/Au. The hole mobility was calculated to be 7.38 × 10^−4^ and 5.54 × 10^−5^ cm^2^ V^−1^ s^−1^ for 79 and 82, respectively. The higher hole mobility of 79 was originates from the efficient ICT and prevented aggregation in solid-state. Furthermore, increased PL quenching behavior of the derivatives in the order of 79 > 82, which ensures better performance of 79. To evaluate the electronic properties of the molecules, PSC was fabricated in the configuration of FTO/TiO_2_/C-PCBSD Perovskite/79 or 82 (100 nm) or spiro-OMeTAD (150 nm)/Au. Where C-PCBSD was crosslinked [6,6]-phenyl-C_61_-butyric styryl dendron ester. Among them, the best performance was observed for 79 with *J*_sc_ of 20.3 mA cm^−2^, PCE of 16.4%, and a FF of 73.5%, which are lesser than the classic spiro-OMeTAD molecule.

Very recently, Grätzel^52^ and the team reported HAB appended triarylamine HTM for PSC. The metal-catalyzed cyclotrimerization of triarylaminealkynes yielded derivative 83, with six oligotriarylamine segments. The absorption spectrum of molecule 83 was limited in the UV region, which is desirable for solar cells (Fig. 18). Molecule 83 was showed thermal stability up to 442 °C, which ensures better durability. HOMO and LUMO of the molecules are estimated to be −5.05 and −1.96 eV. To evaluate its behavior as HTM, 83 was fabricated in mesoporous PSC device with the configuration of FTO/compact TiO_2_/mesoporous TiO_2_/Cs_0.5_(MA_0.15_FA_0.85_)_0.95_Pb(I_0.85_Br_0.15_)/83/Au, where Cs_0.5_(MA_0.15_FA_0.85_)_0.95_Pb(I_0.85_Br_0.15_) is triple-cation perovskite. The device showed PCE of 16.77% and the enhanced performance observed for the device with additive. While using tris(2-(1*H*-pyrazol-1-yl)-4-tertbutylpyridine)cobalt(iii)-tri[bis(trifluoromethane)-sulfonimide] (FK209) as additive the FF increased to 70–72%, consequently PCE improved to 17.48%. This is due to the additional FK209 was reduced the energy level of 83 by oxidizing HTM. This also increasing *V*_oc_ by increasing the energy gap between the HOMO of 83 and Fermi level of TiO_2_, respectively. Consequently, it was reduced the energy barrier of perovskite and 83, which led to increased PCE. Thus, the approach on easy and versatile chemistry developed a successful molecule for PSC at low-cost with enhanced performance.

### 4.3. Miscellaneous electronic applications

HAB and HPB derivatives have been shown interesting properties in OLED and OSC devices and they also have a significant number of reports with quality performances in other electronic devices such as memory devices, logic devices, artificial photosynthesis, and so on.

#### 4.3.1 Memory devices

In the age of information sciences, the mandate for the improvement of innovative data storing materials has been massive. Storage capacity and faster memory structures are the efficiency measures for memory devices.^149^ To gain these needed properties in reasonable values needs much of cost. Organic memory devices (OMDs) came as an ultimate key solution. Though many organic molecules have been used for OMDs, the report by Ming,^21^ explains the application of ruthenium(ii) complexes which employed in donor–π–acceptor–π–donor (DπAπD) type molecular system. Herein, bipyridine appended triarylamine ligand connected to polyphenylene moiety, where polyphenylene moiety is equipped as an electron donor and central Ru-biphenyl segment served as acceptor. The molecule was designed with, the heavy metal center to improve the electroluminescence quantum yield of the molecule. Furthermore, this system helps to improve the spin–orbit coupling, thus increasing the intersystem crossing and permitting the collection of triplet and singlet excitons. The nanosecond transient absorption spectra reveal that the PPB appended triphenylamine increased the π-extended system and lowers the HOMO–LUMO energy difference of the molecule. Due to promising results, 84 has been utilized in the fabrication of organic resistive memory devices using the configuration of ITO/84/Al. The active layer (84) was obtained with the ON/OFF current ratio of over 10^4^ (Fig. 18). The device was stable under a given voltage (electrical energy) bias because it could be switched to the ON state again after the OFF state, which was confirmed by repeating the experiment three times. This memory characteristic is attributed to the electron denser electron-donor portion is completely transferred to the electron-acceptor portion under an electric field, which is results to produce a constant charge-separated state. This is known as the “static random-access memory” (SRAM) type memory characteristics. These results propose the possibility of these compounds as promising candidates for the fabrication of SRAM-type memory devices.

#### 4.3.2 Sequential logic devices

Sequential logic is an important element in electrical circuits. Molecular analogs of this operation have been established throughout the last 20 years, such as sensors for detecting toxic elements. Sequential logic devices are a class of digital electronic devices that are different from the combinational logic devices, which alter the state reliant on the signals given to their inputs at that moment. The sequential logic circuits are different in the way that they can take into description their earlier input signals along with those existent. It has some form of inherent “Memory function”. This memory function remembers the outcome of the previous input. The memory element is recognized as a feedback loop, whose paths are “cyclic” in nature. Sequential circuit alterations happen merely on the utilization of a clock signal to create asynchronous nature. Otherwise, the circuit path is asynchronous and relies on outside input. The memory element of the sequential logic devices can be established using chemical inputs and outputs.

**Fig. 19:**
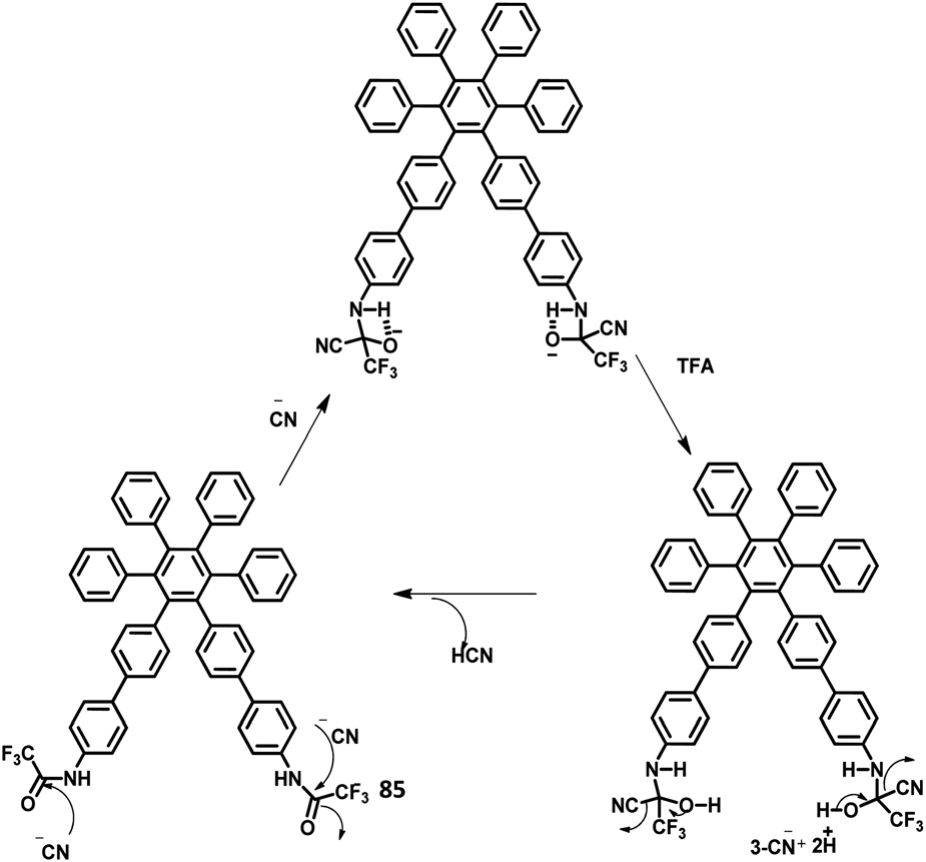
Possible mechanism of reaction based HAB 85 with CN^−^ and TFA.

The work reported by Pramanik *et al.*^150^ describes the use of AIEE-active HPB-based fluorophore for the recognition of CN^−^ ions at the nanomolar concentration using sequentially functioning logic circuits. The florescent spherical nano aggregates of 85 work as a reversible reaction rooted system for the ratiometric detection of cyanide ions and TFA/H^+^. The cyanide ions were detected in the range of ∼2.6 ng cm^−2^ concentration by using 85 coated paper strips, which offer a low-cost method for the detection of CN^−^ in the aqueous medium (Fig. 19). It duplicates the function of a set–reset remembered sequential logic circuit through the input signals of the cyanide and TFA/H^+^ ions (up to pH ≤ 3).

## 5. Concluding remarks and outlooks

During the past decade, star-shaped hexaarylbenzene derivatives were extensively studied for application in the field of synthetic and material chemistry. The non-planar structure and low degree of self-assembly of HAB molecules render amorphous packing, which is exceedingly favorable in optoelectronic devices. Due to their extended conjugation and electron-donating ability, HABs can be engineered with appropriate acceptor moieties to yield push–pull systems. Besides, HAB molecules possess a wider energy gap and higher HOMO energy levels, which are favorable for electronic applications. Thus, these molecules can be used as hole/electron transport materials in organic electronic devices. The stable emission, high quantum efficiency, and high triplet excited energy levels offered by the HAB derivatives make them promising materials for fluorescent and electrophosphorescent light-emitting diodes. The techniques such as AIDF, TADF, and exciplex formation of HAB derivatives are extremely potential to design long lifetime, high efficiency, pure emission chromaticities, eco-friendly, and cost-efficient devices for commercial light-emitting applications. The HAB derivatives with anchoring templates were developed for OSC and perovskite solar cells to convert harvested solar energy to electrical energy. Furthermore, a significant number of reports showed the potential application of new π conjugated HAB derivatives in other electronic devices such as memory devices, sequential logic gates, and artificial photosynthesis. The structure–property relationship is useful to find the ideal derivatives for molecular electronic devices. New and simple synthetic methodologies were developed to synthesize symmetric and unsymmetric polyarylbenzene derivatives. The HAB derivatives are mainly synthesized *via* Diels–Alder cycloaddition reaction, Co-catalyzed cyclotrimerization, and Pd-catalyzed coupling reactions. The developed synthetic methods for desirable substitutions allow the use of HAB-based derivatives in molecular electronics.

Even 80 years after the synthesis of hexaarylbenzene, the unpredictable geometry of the molecule has promoted its research over the decades. There have been many research groups investigating the stereochemistry and geometry of hexaarylbenzene. It is an intermediate of graphene analogs finding immense applications in material chemistry. Apart from the works mentioned in this review, there have been extensive studies on HABs that render interesting information about their applications. These molecules can be potentially considered for future applications such as energy materials for batteries, semiconductors for organic-light emitting transistors, and nanoribbons in graphene chemistry.

## Conflicts of interest

There are no conflicts to declare.

## Supplementary Material
